# Measuring the Time-Scale-Dependent Information Flow Between Maternal and Fetal Heartbeats During the Third Trimester: Impact of Fetal Sex and Maternal Chronic Stress

**DOI:** 10.3390/biology15100749

**Published:** 2026-05-09

**Authors:** Nicolas B. Garnier, Maria S. Molinet, Marta C. Antonelli, Silvia M. Lobmaier, Martin G. Frasch

**Affiliations:** 1 CNRS, ENS de Lyon, LPENSL, UMR5672, 69342 Lyon, Cedex 07, France; 2 Department of Obstetrics and Gynecology, TUM Hospital, School of Medicine, Technical University of Munich, 81675 Munich, Germany; 3 Instituto de Biología Celular y Neurociencia “Prof. E. De Robertis”, Facultad de Medicina, Universidad de Buenos Aires, Buenos Aires 1121, Argentina; 4 Department of Obstetrics and Gynecology, School of Medicine, University of Washington, Seattle, WA 98195, USA; 5 Institute on Human Development and Disability, School of Medicine, University of Washington, Seattle, WA 98195, USA

**Keywords:** multiscale analysis, information theory, high-order statistics, fetal heart rate, causality

## Abstract

Prenatal maternal stress alters maternal–fetal heart rate coupling, as demonstrated by the Fetal Stress Index derived from bivariate phase-rectified signal averaging. Here, we extend this framework using information-theoretical measures to elucidate underlying mechanisms. In 118 third-trimester pregnancies (59 stressed, 59 control), we computed entropy rate (ER), sample entropy (SE) and transfer entropy (TE) under multiple conditioning paradigms, employing mixed linear models for repeated measures. We identified dual coupling mechanisms at the short-term (0.5–2.5 s), but not long-term (2.5–5 s) time scales: (1) stress-invariant state-dependent synchronization, with maternal decelerations exerting approximately 60% coupling strength on fetal heart rate complexity—a fundamental coordination conserved across demographics; and (2) stress-sensitive temporal information transfer (TE), showing exploratory associations with maternal cortisol that require replication. A robust sex-by-stress interaction emerged in TE from mixed models, with exploratory female-specific coupling patterns absent in males. Universal acceleration predominance was observed in both maternal and fetal heart rates, stronger in fetuses and independent of sex or stress. We provide insight into the dependence of these findings on the sampling rate of the underlying data, identifying 4 Hz, commonly used for ultrasound-derived fetal heart rate recordings, as a sufficient sampling rate to capture the information flow. Information-theoretical analysis reveals that maternal–fetal coupling operates through complementary pathways with differential stress sensitivity, extending the Fetal Stress Index by elucidating causal foundations. Future studies should explore additional information-theoretical conditional approaches to resolve stress-specific and time-scale-specific differences in information flow.

## Simple Summary

Understanding how maternal and fetal hearts communicate during pregnancy is crucial for identifying fetuses at risk from prenatal stress. We investigated the directional flow of information between maternal and fetal heart rates in 118 mother–fetus pairs during the third trimester, comparing pregnancies affected by psychosocial stress (n = 59) to controls (n = 59). Using transfer entropy analysis across multiple time scales, we discovered two distinct coupling mechanisms: a stress-invariant synchronization where maternal heart rate decelerations influence fetal heart rate complexity at approximately 60% of the mother’s own self-regulatory strength, and a stress-sensitive information transfer mechanism that shows sex-specific vulnerability in male fetuses from stressed pregnancies. Both maternal and fetal hearts predominantly accelerated together, regardless of stress exposure. Importantly, we established that standard 4 Hz fetal monitoring equipment provides sufficient temporal resolution for detecting these physiological interactions. These findings reveal how prenatal stress disrupts maternal–fetal cardiac communication in a sex-dependent manner, potentially explaining differential developmental outcomes. This knowledge advances our understanding of fetal programming mechanisms and may inform clinical strategies for identifying and protecting vulnerable fetuses during pregnancy.

## 1. Introduction

Intermittent coupling of maternal and fetal heartbeats has been established [1,2,3,4]. Maternal–fetal heartbeat synchronization can serve as a biomarker of chronic stress during pregnancy, impacting maternal and fetal as well as postnatal well-being [5]. Such findings are not only of scientific interest but also clinically actionable during pregnancy, helping increase awareness of chronic stress and inform behavioral and social interventions to reduce it [5,6]. In addition, there is evidence of sex-specific effects of chronic stress [7,8]. However, much remains unclear about this phenomenon [1]. Specifically, its developmental physiology and the methodological and information-theoretical underpinnings of its computation remain active areas of research.

Here, we sought to apply the transfer entropy (TE) [9,10,11] approach to elucidate the dynamics of the information flow between the mother and the fetus. Given the rising interest in wearables, which typically sample heart rate at low sampling rates to monitor wellness and clinical characteristics, we also sought to investigate the relationship between various TE-derived metrics and attributes of data acquisition and processing, with a focus on sampling rate and postnatal health outcomes. For this, we focused on the acceleration- and deceleration-dependent TE metrics.

## 2. Methods

### 2.1. Cohort Characteristics

This prospective cohort study enrolled pregnant women receiving prenatal care between June 2016 and July 2019. Inclusion criteria were singleton pregnancy, gestational age of 32–40 weeks at enrollment, and absence of known fetal anomalies. The study was approved by the local IRB (“Ethikkommission der Fakultät für Medizin der Technischen Universität München”, registration number 151/16S, ClinicalTrials.gov registration number NCT03389178), and all participants provided written informed consent. The complete experimental design is reported in [1].

Maternal stress exposure was assessed using the Perceived Stress Scale (PSS) [12] and the Prenatal Distress Questionnaire (PDQ) [13]. Participants were classified as “stressed” (PSS ≥ 19) or “control” (PSS < 19) based on established cutoffs for elevated perceived stress. Maternal hair cortisol concentration (pg/mg) was measured as a physiological marker of chronic stress exposure.

The initial findings of the underlying FELICITy study, using the bPRSA approach and the SAVEr algorithm for fetal/maternal ECG and R peak extraction, have been reported elsewhere [1,6,14].

**Figure 1:**
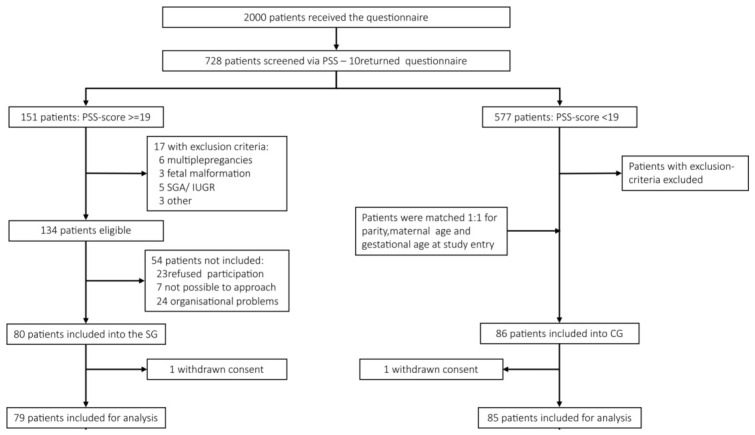
Recruitment flow chart for the FELICITy dataset. The flowchart depicts the original cohort of 164 screened participants (79 stressed, 85 controls). After removal of 47 dyads (45 due to fHR indistinguishable from mHR, 1 dyad lacking clinical records, and 1 dyad with a missing PSS score precluding stress classification), 118 mother–fetus dyads with complete clinical and entropy data were retained for analysis (59 stressed, 59 controls; 49 male, 69 female fetuses). One participant (FS-124) was additionally excluded from Table 4 only (n = 117) due to an extreme outlier net TE value (z-score = 23.7).

Stressed mothers were matched 1:1 with controls on parity, maternal age, and gestational age at study entry. Recruited subjects were between 18 and 45 years of age and were in their third trimester. The subjects were selected from a cohort of pregnant women followed in the Department of Obstetrics and Gynecology at “Klinikum rechts der Isar” of the Technical University of Munich (TUM). This is a tertiary center of Perinatology located in Munich, Germany, which serves 2000 mothers/newborns per year. Figure 1 presents the recruitment flowchart for this dataset and the data used in this study.

Four exclusion criteria were applied, namely, (a) serious placental alterations defined as fetal growth restriction according to Gordijn et al. [15]; (b) fetal malformations; (c) maternal severe illness during pregnancy; (d) maternal drug or alcohol abuse.

The Cohen Perceived Stress Scale questionnaire was administered to gauge chronic non-specific stress exposure (PSS-10) [12]. PSS-10 ≥ 19 categorized subjects as stressed, as established [1]. The PSS-10 is a 10-item instrument designed to measure the degree to which situations in one’s life are appraised as stressful. PSS items were designed to tap the degree to which respondents found their lives unpredictable, uncontrollable, and overloading. We applied inclusion and exclusion criteria following the return of the questionnaires. When a subject was categorized as stressed, the next screened participant, matched on gestational age at recording and with a PSS-10 score <19, was entered into the study as a control. In addition to PSS-10, the participants received the German Version of the “Prenatal Distress Questionnaire” (PDQ), which contains 12 questions on pregnancy-related fears and worries regarding changes in body weight and other pregnancy-related issues, the child’s health, delivery, and the impact of pregnancy on the woman’s relationship.

### 2.2. Fetal and Maternal Heart Rate Acquisition

Briefly, in this study, we used the mHR and fHR data extracted previously in this dataset using the fetal and maternal ECG deconvolution algorithm SAVER [14].

SAVER extracted fetal ECG (fECG) and maternal ECG (mECG) at a 1000 Hz sampling rate from the raw transabdominal ECG (aECG). The aECG signal contains the simultaneous fetal and maternal ECG recordings. The fetal and maternal RR interval time-series were subsequently derived from the fetal and maternal R-peaks. We then calculated the fetal heart rate (fHR) and maternal heart rate (mHR) values. We calculated the signal quality index (SQI) [14] for aECG in 1 s windows and subsequently discarded segments with an SQI below 0.5.

The clinical setting was standardized as much as possible across all study participants. The recordings were performed on all women in the supine half-left recumbent position, usually at the same time of day (afternoon). The raw aECG was recorded at a 900 Hz sampling rate for at least 40 min at 2.5 weeks after initial screening. The AN24 (GE HC/Monica Health Care, Nottingham, UK) was used.

Using the $f_{\mathrm{ECG}}=1000$ Hz sampling rate of ECG, we estimated a noise of the order of $\Delta \left(RRI\right)≃2/f_{\mathrm{ECG}}=2.10^{-3}$ s in the R-R interval estimation. For a typical mean heart rate 〈HR〉≃80 bpm, or mean R-R interval 〈RRI〉≃0.75 s, this corresponds to an estimated noise $\Delta \left(HR\right)=60\times \Delta \left(RRI\right)/{\langle RRI\rangle }^{2}={\langle HR\rangle }^{2}\times \Delta \left(RRI\right)/60≃0.2$ bpm in the HR data.

Upon delivery of the baby, we recorded the clinical data, including birth weight, length, and head circumference, pH, and Apgar score, as previously reported [1].

Next, we removed 47 mother–fetus dyads from the cohort: 45 because for these dyads the fHR was of the order of the mHR (i.e., below 100 bpm) for a significant fraction of the experiment, one dyad (FS-004) for which entropy data were computed but no clinical record was available, and one dyad (FS-144) excluded due to a missing PSS score precluding stress group classification.

### 2.3. Low-Pass Filtering

The raw HR signal was low-pass filtered using a moving average over a time window τ corresponding to the time scale under study, then down-sampled at $f_{s}=20$ Hz. We varied τ in [0.5–20 s] to explore the distribution of information in the heart rates. See Supplementary Methods, Section S1.1, for mathematical details and Supplementary Figure S1.

### 2.4. HR Decelerations and Accelerations

#### 2.4.1. Definition of HR Decelerations and Accelerations

Accelerations and decelerations were defined at each time scale τ based on the sign of the time-derivative of the filtered HR signal, using a zero threshold (μ=0) to ensure sufficient data points. The partitioning into acceleration and deceleration epochs was time-scale-dependent, with smaller τ yielding more numerous but shorter events. See Supplementary Methods, Section S1.2, Figure S2, for formal definitions.

#### 2.4.2. Quantification of Acceleration/Deceleration Events

Heart rate accelerations and decelerations were identified for both maternal and fetal HR. For each recording, we computed the total number of acceleration events (N_accel) and deceleration events (N_decel), allowing calculation of event fractions:

- Acceleration fraction = N_accel / (N_accel + N_decel)
- Deceleration fraction = N_decel / (N_accel + N_decel)

These were computed separately for maternal HR conditioning and fetal HR conditioning.

### 2.5. Information, Complexity and Information Flow Between Maternal and Fetal HR

#### 2.5.1. Entropy Metrics: Overview

Three categories of entropy-based features were computed from the low-pass-filtered mHR and fHR time series over a range of time scales τ from 1/fs = 0.05 s up to 20 s, two univariate quantities (entropy rate and sample entropy) and a bivariate quantity (transfer entropy):

**ER**: Entropy rate quantifies the complexity or unpredictability of a time series, representing the rate at which new information is generated. A higher entropy rate indicates greater irregularity in HR dynamics. ER is a univariate metric. It was computed separately for fetal and maternal HR signals.

**SE**: Sample entropy measures the regularity of a time series by quantifying the conditional probability that sequences similar for *m* points remain similar for m+1 points [16,17]. A lower SE indicates more regular, predictable patterns. SE was computed with embedding dimension m=1 and tolerance r=0.2σ, where σ is the standard deviation of the signal under study. SE is also a univariate metric, computed separately for fetal and maternal HR signals.

**Transfer Entropy (TE)**: Transfer entropy quantifies the directed information flow between two time series, measuring the extent to which knowledge of one signal reduces uncertainty about the future of another. TE was computed bidirectionally between maternal and fetal HR.

**Figure 4:**
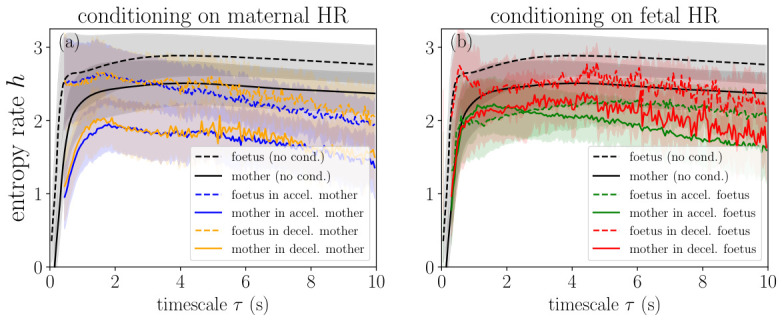
Entropy rate *h* as a measure of information or complexity in the conditioned signals. (**a**) Results when conditioning is performed on the maternal HR signal, and (**b**) when it is performed on the fetal HR signal. Dotted lines pertain to fetal HR and continuous lines to maternal HR. Blue (resp., orange) curves are obtained by conditioning on mHR accelerations (resp., mHR decelerations). Green (resp., red) curves are obtained by conditioning on fHR accelerations (resp., fHR decelerations). For reference, we have plotted the entropy rate measured in fHR (dotted black line) and mHR (continuous black line) without any conditioning. For each curve, a shaded area represents the dispersion of the values over the cohort.

All features were computed using our own toolbox [18]. For each feature *A*, both maximum $A^{\max}$ and mean $A^{\mathrm{AUC}}$ values were extracted using the time-scale interval τ∈[0.5,2.5] s. The maximum value was computed as the maximum of the feature *A* in the interval and was labeled $A^{\max}$ or referred to as max *A*. The mean value was computed as the average of the feature *A* over the interval which, up to a factor 2 s is exactly the area under the A(τ) curve (see Figure 4 for such curves for ER and Supplementary Figure S3 for TE); we label it $A^{\mathrm{AUC}}$ and refer to it as mean *A*.

**Physiology of the chosen time scales**: This time-scale interval, selected based on the range where net TE is consistently positive (Figure S3), aligns with established physiological time constants of autonomic cardiovascular regulation. The lower bound (0.5 s) corresponds approximately to one fetal cardiac cycle (∼0.43 s at 140 bpm) and to the minimum latency of the fast vagal baroreflex arc [19]. The upper bound (2.5 s) encompasses the cardiac vagal baroreflex loop (∼1–2.5 s) [20], while falling below the time scale of sympathetically mediated oscillations such as Mayer waves (∼10 s, corresponding to the low-frequency HRV band at ∼0.1 Hz) [21]. This interval thus predominantly captures parasympathetic (vagal) heart rate modulation, including respiratory sinus arrhythmia (RSA), which in adults operates at respiratory frequencies of 0.15–0.4 Hz (periods of 2.5–6.7 s) with beat-to-beat modulation within each breath cycle [22]. In the fetus, autonomic time constants are less well characterized but generally shorter due to higher resting heart rates; fetal breathing movements, when present, occur at 30–70 breaths/min (periods of ∼0.9–2 s) [23]. The observation that net TE is positive within this vagally dominated time-scale range but not at longer, sympathetically dominated time scales (2.5–5 s) is consistent with maternal–fetal heart rate coupling being mediated primarily through fast, vagal pathways rather than slower sympathetic mechanisms.

#### 2.5.2. Entropy Rate

The entropy rate h(τ) quantifies the rate of new information generation in a signal at time scale τ, computed from the Shannon entropy of the filtered signal and its time-embedded counterpart. For the time-scale range [0.5; 2.5] s, we extracted both the maximal value $h^{\max}$ and the mean value $h^{\mathrm{AUC}}$. See Supplementary Methods, Section S1.3, for formulas.

#### 2.5.3. Transfer Entropy

Transfer entropy (TE) quantifies directed information flow between two signals [24], measuring how much the maternal heart rate history improves prediction of the fetal heart rate future beyond the fetal signal’s own history. TE has been applied to maternal–fetal HR coupling [9,10,11]. We computed TE bidirectionally using a nearest-neighbor estimator (k=5) with surrogate-based bias correction. We report net TE (mother-to-fetus minus fetus-to-mother), as well as TE^max^ and TE^AUC^ in the [0.5; 2.5] s range. See Supplementary Methods, Section S1.4, for complete formulas and estimation details.

#### 2.5.4. Conditioning Framework

We computed entropy metrics at three levels: (1) univariate baseline (no conditioning), (2) self-conditioned (signal during its own events), and (3) cross-conditioned bivariate (signal during the other signal’s events). Each quantity was computed under five conditioning paradigms, yielding 50 entropy-based features in total (20 ER + 20 SE + 10 TE). See Supplementary Methods, Section S1.5, for complete feature enumeration.

### 2.6. Neurodevelopmental Outcome Assessment

Infant neurodevelopmental outcomes were assessed at 24 months using the BSID-III (Bayley Scales of Infant Development III). Composite scores were obtained for cognitive (COG), language (LANG), and motor (MOTOR) domains [25]. Subscale scores for language (receptive, expressive) and motor (fine, gross) skills were also analyzed.

### 2.7. Statistical Analysis

#### 2.7.1. Correlation Analysis (TE/ER/SE vs. Clinical Outcomes)

**Normality Assessment**: Distributional properties of all variables were assessed using the Shapiro–Wilk test (α = 0.05). Correlation method selection was data-driven: Pearson product-moment correlation was applied when both variables satisfied normality assumptions; Spearman rank correlation was used otherwise.

**Univariate Correlations**: Bivariate correlations were computed between each entropy feature (TE, ER, SE) and outcome variable (cortisol, Bayley scores, PSS, PDQ). This yielded 144 independent correlation tests across feature–outcome combinations. Given the exploratory nature of this analysis, uncorrected *p*-values are reported with nominal significance threshold of *p* < 0.05 (two-tailed). However, we also report false discovery rate (FDR) corrected q-values using the Benjamini–Hochberg procedure to assess robustness to multiple comparison correction. At the expected false positive rate of 5%, approximately 7.2 spurious significant findings would be anticipated by chance alone. All findings should be interpreted as hypothesis-generating rather than confirmatory and require independent replication.

**Stratified Analyses**: To examine potential moderating effects, correlations were computed separately for: (1) stressed versus control groups based on PSS classification and (2) male versus female fetuses.

#### 2.7.2. Mixed Linear Model Analysis (Repeated Measures)

Mixed linear models (MLMs) with REML estimation were used for three analyses: (1) acceleration/deceleration ratios (472 observations, 118 patients, excluding FS-124 from Table 4 only), (2) entropy rate with conditioning (1006 observations), and (3) sample entropy with conditioning (2348 observations). Random intercepts for Patient_ID accounted for within-subject correlation. All models included two-way interactions. Analyses were conducted in Python 3.9 using statsmodels 0.14.4. See Supplementary Methods, Section S1.6, for complete model specifications.

#### 2.7.3. Sensitivity Analysis

All MLM results were robust to adjustment for gestational age at birth, maternal age, and pre-gestational BMI (maximum coefficient changes: 7% ER, 6% SE, 18% TE; no significance changes). See Supplementary Methods, Section S1.7.

#### 2.7.4. Multivariate Modeling

Multiple regularized regression and dimensionality reduction approaches (Elastic Net, PCA+Ridge, PLS, Random Forest, parsimonious forward selection) were applied to assess whether entropy features jointly predicted neurodevelopmental outcomes. All features were z-score-standardized and evaluated using 5-fold cross-validation. See Supplementary Methods, Section S1.8, for complete methodological details.

## 3. Results

### 3.1. Study Sample Characteristics

After signal pre-processing steps, a total of 118 mother–fetus dyads with complete clinical and entropy feature data were included from the initial 165. The cohort was balanced for maternal stress exposure: 59 participants (50.0%) were classified as controls and 59 (50.0%) as stressed. The sample included 49 male (41.5%) and 69 female (58.5%) fetuses.

Table 1 presents the clinical and demographic characteristics of the 118 included mother–fetus dyads stratified by prenatal stress exposure. The stressed group had significantly higher pre-gestational BMI (median 23.8 vs. 21.5 kg/m^2^, p=0.009), lower educational attainment (55.9% vs. 76.7% university degree, p=0.020), and a higher rate of unplanned pregnancies (39.0% vs. 10.2%, p<0.001). The stressed group also showed a higher prevalence of autoimmune disease (20.3% vs. 6.7%, p=0.034), prior preterm birth (8.5% vs. 0%, p=0.027), and gestational diabetes (15.3% vs. 1.7%, p=0.008), resulting in a higher overall complication rate (20.3% vs. 6.7%, p=0.034). Operative deliveries were more frequent in the stressed group (48.3% vs. 28.8%, p=0.037). Maternal age, gestational age at birth, birth weight, umbilical arterial pH, Apgar scores, and NICU admission rates did not differ between groups. Importantly, neither gestational age nor maternal age—the covariates most relevant to fetal heart rate maturation—differed by stress exposure, supporting their omission from the primary mixed models.

The initial and included groups did not differ significantly on the majority of sociodemographic variables, including maternal age (33.0 ± 4.6 vs. 33.4 ± 4.0 years, *p* = 0.557), education level (66.4% vs. 71.7% university degree, *p* = 0.635), marital status (*p* = 1.000), parity (*p* = 0.102), smoking (*p* = 1.000), or autoimmune disease (*p* = 0.282). Prenatal stress exposure was also comparable between groups, with no significant differences in PSS-10 scores (median 18.5 vs. 11.5, *p* = 0.145), PDQ scores (median 11.0 vs. 9.0, *p* = 0.265), or stress group assignment (49.6% vs. 43.5%, *p* = 0.596). Pregnancy outcomes, including gestational age, birth weight, child sex, and obstetric complications, were likewise similar.

Two variables reached statistical significance: household income >5000€/month was lower in the included group (41.2% vs. 63.0%, *p* = 0.019), and hair cortisol concentrations were lower in included participants (median 78.0 vs. 117.0 pg/mg, *p* = 0.034). Notably, neither of these variables showed a pattern consistent with a stress-related selection bias—if anything, the excluded group had higher income and higher cortisol, suggesting that exclusion was driven by a technical signal quality rather than by systematic sociodemographic or psychobiological factors.

**Table 1:** Clinical and demographic characteristics of the analysis sample by prenatal stress exposure.

Variable	All (n = 118)	Control (n = 59)	Stressed (n = 59)	*p*
*Maternal sociodemographic characteristics*				
Maternal age (years)	33.0 ± 4.6	33.4 ± 3.9	32.5 ± 5.2	0.314
Pre-gest. BMI (kg/m^2^)	22.7 (5.3)	21.5 (3.4)	23.8 (9.1)	0.009 *
University degree	79 [66.4%]	46 [76.7%]	33 [55.9%]	0.020 *
Income > 5000€/mo	49 [41.2%]	30 [50.0%]	19 [32.2%]	0.063
Married	88 [74.6%]	48 [81.4%]	40 [67.8%]	0.138
Primipara	59 [49.6%]	32 [53.3%]	27 [45.8%]	0.465
Unplanned pregnancy	29 [24.6%]	6 [10.2%]	23 [39.0%]	<0.001 *
Smoking	6 [5.0%]	1 [1.7%]	5 [8.5%]	0.114
IVF/ICSI	10 [8.4%]	8 [13.3%]	2 [3.4%]	0.095
Iron supplementation	52 [43.7%]	26 [43.3%]	26 [44.1%]	1.000
*Pre-existing conditions*				
Autoimmune disease	16 [13.4%]	4 [6.7%]	12 [20.3%]	0.034 *
Chronic hypertension	2 [1.7%]	1 [1.7%]	1 [1.7%]	1.000
Thrombophilia	2 [1.7%]	1 [1.7%]	1 [1.7%]	1.000
Hx preterm birth	5 [4.2%]	0 [0.0%]	5 [8.5%]	0.027 *
Hx hypertensive disorder	1 [0.8%]	1 [1.7%]	0 [0.0%]	1.000
Hx IUGR/SGA	2 [1.7%]	0 [0.0%]	2 [3.4%]	0.244
*Prenatal stress exposure*				
PSS-10 score	18.5 (12.0)	10.0 (5.5)	22.0 (4.0)	<0.001 *
PDQ score	11.0 (10.0)	7.0 (8.0)	15.0 (10.0)	<0.001 *
Hair cortisol (pg/mg)	78.0 (95.0)	64.0 (76.0)	93.0 (115.0)	0.060
*Gestational complications*				
Gestational diabetes	10 [8.4%]	1 [1.7%]	9 [15.3%]	0.008 *
Gestational hypertension	3 [2.5%]	2 [3.3%]	1 [1.7%]	1.000
PE/eclampsia/HELLP	1 [0.8%]	0 [0.0%]	1 [1.7%]	0.496
Any complication	16 [13.4%]	4 [6.7%]	12 [20.3%]	0.034 *
*Birth and neonatal outcomes*				
GA at birth (weeks)	39.7 (1.9)	40.0 (1.4)	39.4 (2.0)	0.200
Birth weight (g)	3533 ± 407	3504 ± 408	3562 ± 407	0.448
Child sex (male)	49 [41.2%]	30 [50.0%]	19 [32.2%]	0.063
Operative delivery	45 [38.5%]	17 [28.8%]	28 [48.3%]	0.037 *
Apgar at 5 min	10 (1)	10 (1)	10 (1)	0.064
Umbilical arterial pH	7.27 (0.13)	7.26 (0.11)	7.28 (0.13)	0.190
NICU admission	5 [4.3%]	3 [5.1%]	2 [3.5%]	1.000

Continuous variables: mean ± SD (normal) or median (IQR) (non-normal). Categorical variables: *n* [%]. Tests: Welch’s *t*-test, Mann–Whitney *U*, or Fisher’s exact. Any complication: gestational diabetes, chronic or gestational hypertension, PE/eclampsia/HELLP, or IUGR/SGA. Hx = history of; GA = gestational age; PE = preeclampsia. Umbilical arterial pH was measured from cord blood at delivery. * p<0.05.

Neurodevelopmental follow-up data were available for a subset of participants: cognitive composite scores (*n* = 66, 55.9%), language composite scores (*n* = 60, 50.8%), and motor composite scores (*n* = 64, 54.2%). Hair cortisol data were available for 90 participants (76.3%). We compared baseline characteristics between participants with and without Bayley follow-up data (Table 2). No significant differences were found in maternal age, gestational age at birth, PSS score, stress group distribution, fetal sex, or birth weight (all *p*-values > 0.05), suggesting that attrition was not systematically related to these variables.

One additional participant (FS-124) was excluded from Table 4 only due to an extreme outlier net TE value (z-score = 23.7), yielding n = 117 for that analysis (58 stressed, 59 control, 49 male, 68 female).

### 3.2. Decelerations and Accelerations: Dependence on Stress and Time Scale

An important feature of our approach is that decelerations and accelerations are defined using filtered signals and varying the time scale. This is in strong contrast to a more classical definition using the raw heart rate signal—which is piece-wise constant—which would require using a non-zero threshold μ.

First, we explore the distribution of decelerations and accelerations as a function of conditioning approach (on mother or on fetus), exposure, and sex. Next, we explore the information-theoretical properties.

**Figure 2:**
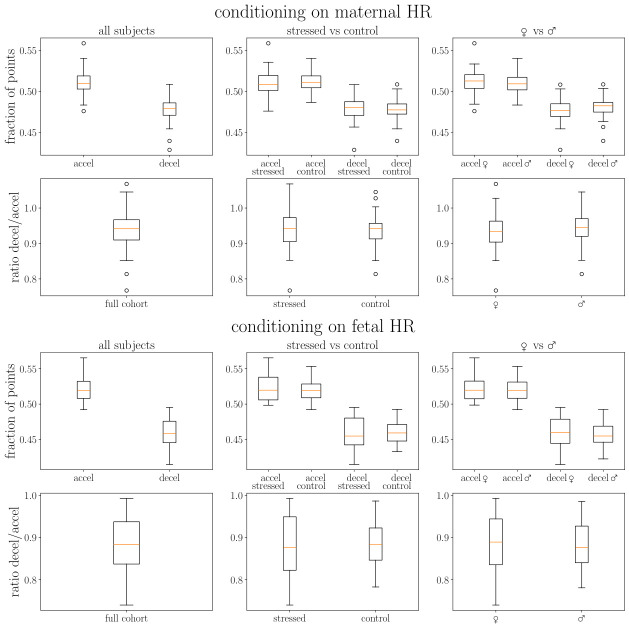
Statistics of deceleration/acceleration ratios computed at time scale τ=2.5 s. The upper and lower parts correspond to the mother’s HR and the fetus’s HR, respectively. In each case, the first line presents the fraction of time points that are decelerations or accelerations. In contrast, the second line presents the ratio of decelerations to accelerations.

**Figure 3:**
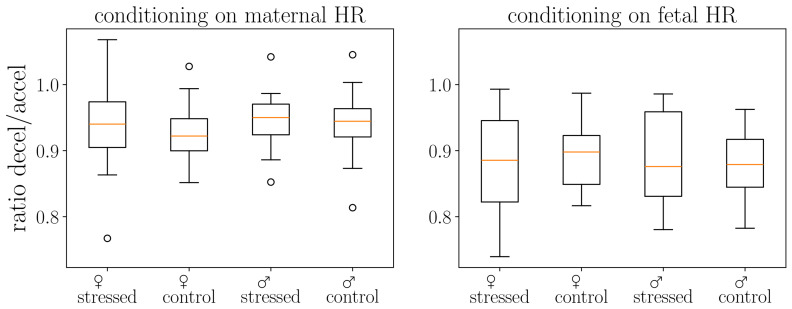
Group dependencies of statistics of deceleration/acceleration ratios computed at time scale τ=2.5 s, for female (♀) and male (♂) fetuses, in both the stressed and control group.

We present in Figure 2 and Figure 3 the distribution of the ratio of decelerations to accelerations in the cohort. The ratios of deceleration/acceleration are always around one. The ratio below one indicates the dominance of accelerations. Interestingly, we observe slightly fewer decelerations than accelerations, especially for fetal heart rates, consistent with previous literature [26].

#### 3.2.1. Overall Patterns

Mixed linear model analysis revealed a significant main effect of event type (β = −0.061, SE = 0.0028, *p* < 0.001) and HR_Source × Event_Type interaction (β = 0.028, SE = 0.0028, *p* < 0.001).

Accelerations were significantly more common than decelerations across all conditions. Asymmetry varied by heart rate source (Figure 2):

- Fetal HR: 52.1% accel. vs. 46.0% decel. (6.1% difference)
- Maternal HR: 51.1% accel. vs. 47.8% decel. (3.3% difference)

HR_Source showed a main effect (β = −0.011, *p* < 0.001), i.e., mHR showed a different baseline pattern.

#### 3.2.2. Group Comparisons

We observed no significant demographic effects (Figure 3):

- Sex (male): β = −0.0026, *p* = 0.361
- Stress (stressed): β = +0.0007, *p* = 0.802
- Sex × Stress interaction: β = −0.0001, *p* = 0.985

Acceleration/deceleration asymmetry represents a universal biological phenomenon, consistent across demographic groups in this third-trimester cohort. Full statistical results are presented in Supplementary Tables S1 and S5.

### 3.3. Univariate Information Measures of fHR and mHR: Entropy Rate and Sample Entropy

#### 3.3.1. Identification of Time Scales

The entropy rate was roughly constant across time scales above 0.5–1 s (Figure 4), with lower values below this range reflecting the absence of information between consecutive R-peaks. Unconditioned entropy rate was always greater than conditioned values. Fetal HR generally had higher entropy rate than maternal HR, except when conditioning on fetal accelerations. See Supplementary Results, Section S2.1, for detailed interpretation.

#### 3.3.2. Stress and Sex Effects

**Figure 5:**
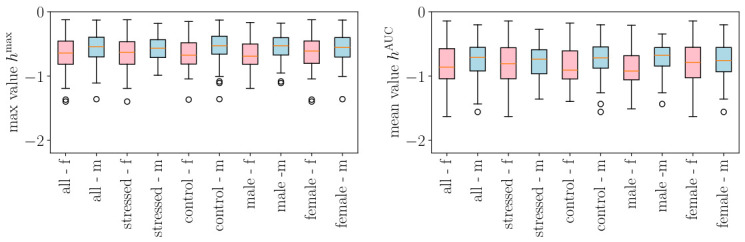
Typical dependence of the maximal value $h^{\max}$ of entropy rate (**left**) as well as of its mean value $h^{\mathrm{AUC}}$ (**right**) in the range [0.5–2.5] s for the mHR (blue—m) and the fHR (pink—f) across the cohort, as well as in each subgroup (stressed or control, female or male fetus).

We represent in Figure 5 how the maximal value $h^{\max}$ (left) of the entropy rate and its mean value $h^{\mathrm{AUC}}$ (right) are distributed, for both mHR and fHR. When the entire signal is considered, i.e., when no conditioning on accelerations or decelerations is performed, the entropy rate seems to always have a very slightly higher value in the mHR than in the fHR; this is observed for all subgroups (stressed and control, as well as male or female fetus).

Because male and female fetuses have different maturation curves, we first compared baseline (unconditioned) fHR entropy between male (n = 49) and female (n = 69) fetuses. There were no significant sex differences in either fetal entropy rate measure: $h^{\max}$ (males: −0.67±0.24 vs. females: 0.63±0.27; t = −0.69, *p* = 0.490, Cohen’s d = −0.13) or $h^{\mathrm{AUC}}$ (males: −0.85±0.30 vs. females: −0.80±0.34; t = −0.90, *p* = 0.370, Cohen’s d = −0.17). Maternal heart rate entropy likewise did not differ by fetal sex (both *p* > 0.57).

Importantly, these baseline findings are consistent with the mixed linear model results reported in Section 3.2.2: the main effect of sex was non-significant across all three models, acceleration/deceleration fractions (β=0.003, *p* = 0.361), entropy rate (β=−0.106, *p* = 0.177), and transfer entropy (β=+0.018, *p* = 0.113). The significant findings emerged exclusively at the level of Sex×Stress interaction in the transfer entropy model (β=−0.042, *p* = 0.009), confirming that the observed sex-dependent effects reflect differential stress-related modulation of maternal–fetal coupling rather than pre-existing sex differences in baseline cardiac autonomic complexity.

**Figure 6:**
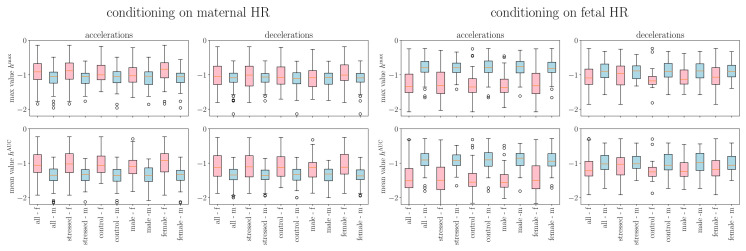
Same as Figure 5 but when considering either accelerations of decelerations, computed on either maternal or fetal HR.

We performed the same analysis on the conditioned signals by examining mHR and fHR computed in either fetal or maternal accelerations or decelerations. Box plots are represented in Figure 6. Sample entropy box plots are not shown here for brevity but as Figures S7 and S8 in the Supplementary Materials. The findings are similar to those of the entropy rate.

Mixed linear model analysis of entropy rate with conditioning framework revealed both univariate signal properties and bivariate maternal–fetal coupling (Figure 5 and Figure 6, Supplementary Table S2).

**Conditioning Effects: Coupling Strength Quantification**

The magnitude of maternal–fetal coupling can be derived from the MLM β coefficients (see Supplementary Box (Section S4 and Table S6) for details). The univariate baseline (no conditioning) represents fetal HR entropy when measured independently (β = +0.206, *p* < 0.001 relative to the reference level of fetal acceleration conditioning). Cross-conditioning on maternal deceleration events reduces entropy by β=−0.123 (*p* = 0.012). The ratio of these coefficients quantifies coupling strength:

$$\mathrm{Coupling}\mathrm{strength}=\frac{|\beta \;\;\mathrm{maternal}\;\mathrm{decelerations}|}{\beta \;\;\mathrm{no}\;\mathrm{conditioning}}=\frac{0.123}{0.206}=0.597\approx 60\%$$

This indicates that the maternal deceleration coupling effect captures 60% of the dynamic range established by the no-conditioning baseline. The coupling effect (β=−0.123) represents a substantial constraint on fHR complexity; the fHR becomes more predictable and regular during mHR decelerations. This 60% reduction is stress-invariant (*p* = 0.128 for stress effect on conditioned entropy), indicating it represents a fundamental physiological coordination mechanism present across all mother–fetus pairs, regardless of maternal stress status.

This is the strongest signature of state-dependent bivariate coupling in our results.

We also observed fetal deceleration conditioning: β=−0.082, SE = 0.042, *p* = 0.054, showing a marginal trend toward entropy reduction.

Comparative coupling hierarchy:

- Maternal deceleration conditioning: 60% coupling strength (β=−0.123, *p* = 0.012 *)
- Fetal deceleration conditioning: 40% coupling strength (β=−0.082, *p* = 0.054 †)
- Maternal acceleration conditioning: 16% coupling strength (β=−0.034, *p* = 0.494, ns)

Maternal deceleration events exhibited 1.5× stronger coupling than fetal events and 3.8× stronger coupling than maternal accelerations, demonstrating a profound asymmetry in maternal–fetal physiological interdependence. Importantly, this asymmetric pattern was conserved across stressed and control groups, suggesting that it reflects a fundamental maternal–fetal communication architecture rather than a stress-specific adaptation.

The entropy rate and sample entropy exhibited differences in behavior. Hmean was consistently 0.117 units lower than Hmax (Hmean vs. Hmax: β=−0.117, SE = 0.052, *p* = 0.023 *). This reflects different aspects of entropy rate estimation.

**Group Comparisons: Differential Stress Sensitivity**

We detected no significant demographic effects in conditioned entropy:

- Sex (male): β=−0.106, *p* = 0.177
- Stress (stressed): β=−0.085, *p* = 0.128 (ns)
- Sex × Stress: β=+0.108, *p* = 0.223 (ns)

Of note, while TE showed significant stress correlations (r = 0.21–0.31 with cortisol, see Table 5, see Supplementary Table S8 for details), the conditioned entropy rate showed no stress effects (*p* = 0.128). This differential sensitivity (keeping in mind the exploratory nature of the reported correlation findings) reveals that temporal information transfer (TE) is stress-modulated while the state-dependent coupling (conditioned entropy) is stress-invariant.

#### 3.3.3. Entropy Progression Reveals Coupling Hierarchy

Progressive entropy reduction from univariate (no conditioning) to cross-conditioned states demonstrates that conditioning constrains signal complexity, cross-conditioning reveals bivariate coupling, and maternal deceleration events exert the strongest influence on fetal HR predictability. See Supplementary Results, Section S2.2, for the complete hierarchy.

#### 3.3.4. Sample Entropy Analysis

Sample entropy computation yielded adequate non-zero observations (2348 observations, 19.9 per patient; cf. Supplementary Table S3). We assessed whether sample entropy exhibited bivariate coupling signatures similar to those of the entropy rate.

The full MLM analysis revealed strong metric and signal effects: SEmean was consistently 0.188 units lower than SEmax (β=−0.188, SE = 0.008, *p* < 0.001), and maternal HR showed higher sample entropy than fetal HR (β = +0.135, SE = 0.019, *p* < 0.001). However, in contrast to entropy rate, sample entropy did not detect significant coupling from maternal decelerations to fetal HR complexity (β=−0.034, SE = 0.019, *p* = 0.073, ns). The coupling ratio for SE (2.7%) was markedly lower than for ER (60%), confirming that these metrics capture fundamentally different aspects of heart rate dynamics.

Notably, strong HR_source × conditioning interactions were observed: maternal HR during maternal accelerations showed marked SE reduction (β=−0.223, SE = 0.027, *p* < 0.001), indicating that sample entropy is sensitive to *within-signal* state changes but not to *cross-signal* coupling, which is the domain of the entropy rate.

No demographic effects were detected (Sex: *p* = 0.542; Stress: *p* = 0.756; Sex × Stress: *p* = 0.572), consistent with entropy rate findings.

#### 3.3.5. Exploratory Neurodevelopmental Associations

Two SE associations with neurodevelopmental outcomes reached nominal significance (*p* < 0.05, uncorrected; Table 3). Neither survived FDR correction (both q = 0.62), consistent with the overall pattern that none of the 144 correlation tests survived multiple comparison adjustment (Supplementary Figure S4).

**Exploratory interpretation**: Sample entropy during fetal HR accelerations showed tentative positive associations with language receptive scores (higher entropy → better receptive language). However, failure to survive FDR correction (q = 0.62) indicates these patterns require replication before drawing biological conclusions.

No significant associations were observed between ER/SE features and maternal stress measures (cortisol, PSS, PDQ), tentatively suggesting distinct pathways whereby TE may capture stress physiology while ER/SE relate to neurodevelopment. However, this dissociation also requires replication, given the overall null FDR-corrected results.

**Sex-Stratified ER/SE Patterns (Exploratory)**

Sex stratification revealed differential ER/SE association patterns (Supplementary Figure S5).

**Female fetuses** (n = 69): In addition to the 16 TE correlations (Section 3.3.3), females showed:

- ER × Motor Gross (four negative correlations, r = −0.37 to −0.61): Fetal ER during various conditioning types showed negative associations with motor gross skills.
- SE × Cognitive/Language (three positive correlations):
  – SE mother full × Cognitive: r = +0.44, *p* = 0.011, n = 33.
  – SE fetus (fHR accel.) × Language Receptive: r = +0.41, *p* = 0.024, n = 30.
  – SE mother (fHR accel.) × Language Receptive: r = +0.41, *p* = 0.024, n = 30.

**Male fetuses** (n = 49): Only the previously noted ER × Motor Composite correlation (r = +0.39, *p* = 0.035).

**Sex × Stress stratification** revealed additional complexity (Supplementary Figure S6):

**Male control**: Strong positive ER/SE × Motor associations dominated (eight ER correlations, two SE correlations with r = +0.49 to +0.72), with the strongest being SE fetus full × Motor Fine (r = +0.72, *p* < 0.001, n=20).

**Female control**: Positive ER × Cognitive/PDQ associations (five correlations, r = +0.38 to +0.65) rather than motor associations.

**Exploratory interpretation**: These sex-stratified patterns (all failing FDR correction with q > 0.40) tentatively suggest sex-differentiated developmental pathways, where males show stronger ER/SE-motor coupling (especially in the control subgroup), while females show broader associations spanning cognitive, language, and motor domains. The strong positive SE–motor associations in male control (r = +0.72) contrast with the minimal ER/SE associations in males overall, suggesting stress may disrupt these pathways. All patterns require replication in larger samples.

### 3.4. Bivariate Information Measures of Information Flow Between fHR and mHR: Transfer Entropy Family

#### 3.4.1. TE and Conditioning

**Figure 7:**
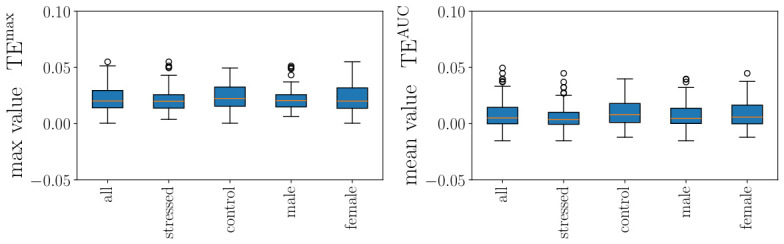
Typical dependence of the maximal value $TE^{\max}$ of the net transfer entropy from mother to fetus, as well as of its mean value $TE^{\mathrm{AUC}}$ in the range [0.5–2.5] s across the cohort (all), as well as in each subgroup (stressed or control, female or male fetus). On the left subplots is also presented the components $TE_{m\to f}$ (mother to fetus) and $TE_{m\to f}$ (fetus to mother) to show their relative order of magnitude: the net TE is smaller than its one-way components.

**Figure 8:**
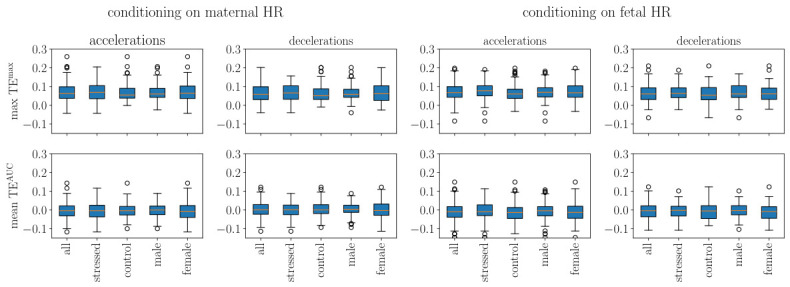
Same as Figure 7 for net TE between the mother and the fetus, but when considering either accelerations of decelerations, computed on either maternal or fetal HR.

In this section, we use the time-scale range [0.5,2.5] s and we compute the maximal values as well as the mean values in this range. We plotted in Figure 7 our findings when using all available points and in Figure 8 when using only accelerations, resp., decelerations, each of which computed using either the maternal HR or the fetal HR. The corresponding statistics for the net TE in Figure 7 and Figure 8 are given in Table 4; they were obtained from a one-sided one-sample *t*-test (positive value versus zero-value) as well as in Supplementary Tables S5 and S9.

**Table 4:** Significance of (a non-vanishing) net transfer entropy (*p*-values).

		TE^max^					TE^AUC^				
		no	mHR	mHR	fHR	fHR	no	mHR	mHR	fHR	fHR
Group	*n*	Cond.	Accel.	Decel.	Accel.	Decel.	Cond.	Accel.	Decel.	Accel.	Decel.
All	117	**0.028 **	0.09	0.09	0.07	0.08	0.25	0.53	0.50	0.57	0.55
Stressed	58	**0.031**	0.10	0.08	0.07	0.07	0.27	0.55	0.53	0.58	0.56
Control	59	**0.024**	0.08	0.09	0.07	0.09	0.23	0.51	0.47	0.56	0.55
Male	49	**0.021**	0.08	0.08	0.09	0.09	0.26	0.52	0.48	0.54	0.52
Female	68	**0.032**	0.10	0.09	0.07	0.08	0.24	0.54	0.50	0.59	0.58

Note. *p*-values represent the probability of observing a zero or negative net TE under a Gaussian assumption fitted to the cohort distribution (see Supplementary Methods, Section S1.8). One participant (FS-124) was excluded from this table due to an extreme outlier net TE value (z=23.7); all other analyses retain this participant. *All* refers to values obtained when using all the cohort. *Stressed*, resp., *control*, refers to values obtained in the stressed, resp., control, subgroup. *Male*, resp., *female*, refers to values obtained in the male fetuses, resp., female fetuses, subgroup. The “no cond.” column name indicates that no conditioning was applied, whereas mHR/fHR and accel./decel. indicate the type of conditioning.

We first comment on the choice of the range of time scales [0.5, 2.5] s: as can be seen in Supplementary Figure S3, this corresponds to the range of time scales where the net TE is expected to be positive, revealing a net information flow from the mother to the fetus. Looking at Figure 7, we then comment on the typical values of the net TE, in contrast to the typical values of TE_*m*→*f*_ between the maternal and the fetal HR and its counterpart TE_*f*→*m*_ between fetal and maternal HR. The one-way information flow from the mother to the fetus is roughly equivalent to the one-way information flow from the fetus to the mother, and the net TE, which expresses the balance between the two, is much smaller: the net flow of information is small, but directed from the mother to the fetus.

Figure 7 shows that when all available points are used, TE^max^ without conditioning is significantly positive across all subgroups (p=0.021–0.032; Table 4), confirming a net information flow from the mother to the fetus. TE^AUC^ without conditioning does not reach significance (p=0.23–0.27). We conclude that a small but significant quantity of information is flowing from the mother to the fetus.

When conditioning is applied, neither TE^max^ nor TE^AUC^ reaches significance (p=0.07–0.10 and p>0.47, respectively; Figure 8 and Table 4), indicating that while the net information flow is consistently positive at the cohort level, its magnitude relative to inter-individual variability is modest. We also observe that max values $TE^{\max}$ are larger if a conditioning (on accelerations or decelerations) is used (Figure 8).

Next, we applied a mixed linear model analysis of transfer entropy values across conditioning types. This approach revealed significant effects of conditioning, TE metric type, and demographic interactions (see Supplementary Table S5 for details).

**Conditioning and Metric Effects**

We detected significant main effects as follows:

- **TE metric type** (AUC vs. Max): β=−0.077, SE = 0.007, *p* < 0.001 ***
  TE^AUC^ was consistently 0.077 units lower than TE^max^ across all conditions. This reflects, intuitively, the different aspects of information transfer quantification via these two quantities.
- **“Baseline” conditioning** (i.e., none): β=−0.037, SE = 0.007, *p* < 0.001 ***
  This represents TE when computed on complete time series, without event-specific conditioning It serves as a reference for conditioned states (accelerations, decelerations).

Importantly, we detected a significant interaction:

- **TE metric × Conditioning interaction**: β = +0.064, SE = 0.010, *p* < 0.001 ***
  This indicates the difference between mean TE^AUC^ and max TE^max^ varies across conditioning types. Unconditioned state (none) shows a larger mean–max differential than event-conditioned states.

**Sex–Stress Interactions**

Unlike the entropy rate, which showed no demographic modulation, TE revealed significant sex–stress interactions:

- **Stress (main effect)**: β = +0.023, SE = 0.010, *p* = 0.026 *
  The stressed group showed higher TE values overall. This is consistent with the here-reported, albeit exploratory, TE–cortisol correlations.
- **Sex × Stress interaction**: β=−0.042, SE = 0.016, *p* = 0.009 **
  The effect of stress differed between male and female fetuses. Male stressed fetuses showed lower TE than expected from additive effects.
- **Sex × Stress × Conditioning interaction**: β = +0.037, SE = 0.015, *p* = 0.014 *
  Three-way interaction indicated that sex–stress differences varied across conditioning types and were most pronounced in the unconditioned (baseline) state.

#### 3.4.2. Differential Stress Sensitivity

Transfer entropy showed significant stress modulation (*p* = 0.026) and sex–stress interactions (*p* = 0.009), contrasting sharply with entropy rate, which showed no stress effects (*p* = 0.128).

This differential sensitivity reveals distinct physiological mechanisms:

- Transfer Entropy: Stress-sensitive (*p* = 0.026)—Temporal information flow modulated by maternal stress.
- Conditioned Entropy Rate: Stress-invariant (*p* = 0.128)—State-dependent coupling robust to stress.

**Biological interpretation**: Stress influences temporal prediction dynamics (how maternal past predicts fetal future) but not instantaneous state dependencies (how concurrent maternal states constrain fetal complexity). This suggests:

- Stress-modulated neural or hormonal pathways affect lagged predictive relationships (TE).
- Fundamental autonomic coordination mechanisms remain robust to acute stress (conditioned entropy).

The significant sex–stress interaction in TE (β=−0.042,p=0.009) indicates that stress effects on temporal coupling differ between male and female fetuses, potentially reflecting sex-specific stress-response pathways or differences in autonomic regulation in utero.

#### 3.4.3. Exploratory Neurodevelopmental Associations of TE

Of 144 correlation tests performed across all entropy features and outcomes, seven uncorrected associations (p<0.05) were identified, matching the expected false positive rate of ∼7.2 at α = 0.05 (Table 5 and Supplementary Figure S4 and Table S4). Critically, none of these associations survived false discovery rate (FDR) correction (all q > 0.40), indicating these findings are exploratory and require independent replication before biological interpretation.

**Table 5:** Transfer entropy associations with maternal cortisol (exploratory).

Feature	r	*p*	q (FDR)	n
Max TE conditioned on mHR decel	+0.315	0.003	0.41	88
Max TE conditioned on mHR accel	+0.287	0.007	0.48	88
Max TE conditioned on fHR decel	+0.271	0.011	0.51	88
Max TE conditioned on fHR accel	+0.250	0.019	0.62	88
Mean TE conditioned on mHR accel	+0.221	0.038	0.68	88

Note. All correlations were computed using the Spearman method. TE = transfer entropy; mHR = maternal heart rate; fHR = fetal heart rate. All q-values > 0.40 indicate failure to survive FDR correction. No other TE–cortisol associations reached p<0.05.

**TE–Cortisol associations**: Five TE–cortisol associations reached nominal significance (*p* < 0.05, uncorrected; Table 5).

**Exploratory interpretation**: All uncorrected significant TE–cortisol associations were positive (r = 0.22–0.31), tentatively suggesting that stronger directional coupling between maternal and fetal heart rates may be associated with higher chronic stress. Maximum TE values showed stronger associations than mean TE values. However, given the failure to survive multiple comparison correction, these patterns require replication.

**TE–Bayley association**: One TE–neurodevelopmental association reached nominal significance (Table 6).

**Exploratory interpretation**: Higher TE during fetal accelerations was tentatively associated with lower expressive language skills. This was the only TE–neurodevelopmental association to reach nominal significance among 30 tests performed, and it did not survive FDR correction (q = 0.73), suggesting a possible Type I error. No significant TE associations were observed with cognitive or motor outcomes, or with psychological stress measures (PSS, PDQ).

#### 3.4.4. Sex-Stratified TE Analysis (Exploratory)

Sex-stratified analysis revealed striking differences in TE–outcome associations, consistent with the robust Sex × Stress × TE interaction from the MLM analysis:

**Female fetuses** (n = 69) exhibited 16 significant correlations (*p* < 0.05, uncorrected), but none survived FDR (all q > 0.40).

Specifically, we observed four TE–cortisol correlations:

- Max TE (fHR accel): r = +0.368, *p* = 0.007, n = 53.
- Max TE (fHR decel): r = +0.373, *p* = 0.006, n = 53.
- Max TE (mHR accel): r = +0.351, *p* = 0.009, n = 54.
- Max TE (mHR decel): r = +0.423, *p* = 0.002, n = 53.

We also observed three negative TE–cognitive performance correlations:

- Max TE (no conditioning): r = −0.410, *p* = 0.018, n = 33.
- Max TE (fHR accel): r = −0.401, *p* = 0.023, n = 32.
- Mean TE (no conditioning): r = −0.377, p = 0.031, n = 33.

**Male fetuses** (n = 49) exhibited only one significant correlation (Entropy rate fetus mHR decel. × Motor Composite: r = +0.387, *p* = 0.035, n = 30), which did not survive FDR correction (q > 0.40). No significant TE–cortisol or TE–cognitive performance correlations were seen in males.

**Exploratory interpretation**: Female-specific maternal–fetal coupling mechanisms may underlie the robust Sex × Stress × TE interaction. In females, higher TE tentatively associates with both higher maternal stress (cortisol) and lower cognitive scores, suggesting that increased directional coupling under stress may reflect compensatory or maladaptive physiological responses. The absence of TE correlations in males tentatively suggests sex-differentiated autonomic regulation pathways. However, all correlation findings failed FDR correction and require replication in adequately powered studies.

#### 3.4.5. Sex × Stress Interaction Patterns (Exploratory)

Further stratification by sex and stress status revealed complex interaction patterns (Supplementary Figure S6):

Male-control fetuses (n = 30) exhibited 16 significant correlations (*p* < 0.05, uncorrected; all q > 0.40):

- Strong positive ER/SE × Motor associations (r = +0.49 to +0.72).
- Positive Mean TE × PSS associations (r = +0.43 to +0.45).
- Positive Mean TE × Cognitive (r = +0.48).

Male-stressed fetuses (n = 19) showed seven significant correlations (all q > 0.40):

- Pattern reversal with negative Max TE × PSS (r = −0.49, *p* = 0.032).
- Negative Mean TE × Language scores (r = −0.65 to −0.70).
- Motor associations absent.

Female-control fetuses (n = 32) exhibited twelve significant correlations (all q > 0.40):

- Strongest TE–cortisol coupling: Max TE (mHR decel) r = +0.55, *p* = 0.006.
- Very strong negative TE × Language: Max TE (fHR accel) × Lang Composite r = −0.86, *p* < 0.001.
- Positive ER × Cognitive/PDQ (r = +0.38 to +0.65).

Female-stressed fetuses (n = 39) showed seven significant correlations (all q > 0.40):

- Weaker TE–cortisol coupling (r = +0.38 vs. r = +0.55 in control).
- Shift from TE to SE/ER dominance for cognitive/language outcomes.
- Positive SE mother × Cognitive (r = +0.54, *p* = 0.014).

**Exploratory interpretation**: These Sex × Stress patterns (all requiring replication due to FDR failure) tentatively suggest that:

1. In **males**, stress eliminates positive motor associations and reverses the coupling–stress relationship direction;
2. In **females**, stress weakens maternal–fetal coupling but activates alternative signal complexity pathways (SE/ER).

These exploratory stratified findings provide potential mechanistic context for the robust Sex × Stress × TE interaction, though the specific correlation patterns require validation in larger samples.

### 3.5. Multivariate Modeling

Multivariate analysis using regularized regression and dimensionality reduction approaches was severely limited by the unfavorable sample-size-to-predictor ratio (n/k≈1.2 vs. recommended >10–20). Cross-validated $R^{2}$ values were predominantly negative, indicating overfitting despite regularization. Feature selection patterns suggested potential domain-specific relevance (TE for motor/cognitive, ER for language) but lacked statistical reliability. Univariate and MLM analyses remain the most interpretable for this sample size. Complete multivariate results are presented in Supplementary Results, Section S2.3.

### 3.6. Methodological Insights: Dependence of TE on HR Sampling Rate

We validated that TE estimates were independent of the HR sampling rate $f_{s}$ (tested at 4, 10, 20, 100, and 1000 Hz), confirming that the standard 4 Hz used for ultrasound-derived fetal HR is sufficient to capture the information flow. For all results reported here, we used $f_{s}=20$ Hz. See Supplementary Results, Section S2.4, for detailed analysis.

## 4. Discussion

### 4.1. Summary of Key Findings

Maternal–fetal heart rate coupling has emerged as a window into prenatal physiological communication, with growing evidence that disruptions in this coupling associate with adverse developmental outcomes [9,10,11]. Building on this literature and on the Fetal Stress Index framework [1], we applied information-theoretical measures to dissect the mechanisms underlying maternal–fetal coupling. Our findings demonstrate that prenatal maternal stress influences specific aspects of physiological communication between mother and fetus, while other fundamental coupling mechanisms remain conserved.

**Principal findings:**

First, we identified dual coupling mechanisms operating simultaneously during the third trimester: temporal information transfer (quantified by transfer entropy) and state-dependent synchronization (quantified by conditioned entropy). These complementary measures reveal that maternal–fetal coupling involves both time-lagged predictive relationships and concurrent state dependencies.

Second, we discovered differential stress sensitivity in these coupling pathways. While temporal information transfer shows stress-related modulation and exploratory associations with maternal cortisol, state-dependent coupling remains stress-invariant—a fundamental physiological coordination that persists regardless of maternal stress status.

Third, our analyses revealed profound asymmetry in maternal–fetal coupling. Maternal heart rate decelerations exert substantially stronger influence on fetal heart rate complexity than any other physiological state, reducing fetal entropy by approximately 60%. This asymmetric coupling may reflect critical regulatory states where maternal–fetal coordination is most pronounced.

Finally, exploratory analyses suggested sex-differentiated coupling patterns, with female fetuses exhibiting stronger temporal coupling associations than male fetuses. These sex-specific patterns require replication but align with emerging evidence of sexually dimorphic fetal autonomic development.

We contextualize these findings within the broader literature on prenatal stress programming, maternal–fetal physiological communication, and autonomic nervous system development.

### 4.2. From BPRSA to Information Theory: Deepening the Fetal Stress Index Framework

Our previous work [1] established the Fetal Stress Index (FSI) using bivariate phase-rectified signal averaging (BPRSA) to quantify maternal–fetal heart rate coupling. That study revealed a fundamental observation: while control fetuses remained physiologically “stable” during maternal breathing cycles, stressed fetuses exhibited fetal heart rate decreases in response to maternal heart rate decreases. The FSI successfully discriminated between stressed and control groups and correlated with maternal perceived stress, validating the concept that maternal stress alters the fetal autonomic response to maternal physiological fluctuations.

The current study builds upon this foundation by applying information-theoretical measures to elucidate the mechanisms underlying these coupling phenomena. Where BPRSA captures phase-rectified signal relationships, transfer entropy quantifies directional information flow, specifically, how the past maternal heart rate improves prediction of the future fetal heart rate beyond the fetal signal’s own history. Where BPRSA identifies average signal responses, entropy rate conditioning reveals how signal complexity changes during specific physiological states, offering insights into state-dependent coupling mechanisms.

Ref. [1]’s observation that stressed fetuses show heart rate decreases during maternal deceleration events finds theoretical grounding in our conditioning framework. The substantial entropy reduction during maternal decelerations—approximately 60% compared to baseline—demonstrates that fetal heart rate becomes highly constrained and predictable during these maternal states. This state-dependent synchronization represents the information-theoretical substrate of the coupling captured by BPRSA.

Critically, we extend the FSI framework by demonstrating that coupling operates through dual pathways. The stress-invariant state-dependent coupling (conditioned entropy) may represent the fundamental physiological coordination mechanism that exists across all mother–fetus pairs, while the stress-sensitive temporal coupling (transfer entropy) may capture the “over-sensitization” hypothesized in our previous work, where maternal stress alters the fetal autonomic system’s temporal response dynamics to maternal physiological changes.

### 4.3. Biological Foundations of Maternal–Fetal Coupling: Literature Context

#### 4.3.1. Prenatal Stress Programming and the HPA Axis

The fetal programming hypothesis posits that prenatal environmental conditions, including maternal psychological stress, can produce lasting alterations in fetal development through multiple pathways [27,28]. Maternal stress activates the hypothalamic–pituitary–adrenal (HPA) axis, elevating cortisol levels that can cross the placental barrier despite partial 11β-HSD2 enzymatic protection [29,30]. Our previous finding of 63% higher hair cortisol concentrations in stressed mothers [1] confirmed chronic HPA axis activation in our cohort.

Elevated fetal glucocorticoid exposure can program the developing HPA axis and autonomic nervous system, altering set points for physiological regulation [31,32]. The exploratory associations between transfer entropy and maternal cortisol observed in our study—while not surviving correction for multiple comparisons and requiring replication—align with this programming framework. They suggest that chronic maternal stress may alter the temporal dynamics of maternal–fetal heart rate coupling, potentially reflecting modified autonomic responsiveness in the developing fetus.

#### 4.3.2. Autonomic Nervous System Maturation

The fetal autonomic nervous system undergoes rapid maturation during the third trimester, with progressive increases in parasympathetic tone and heart rate variability [3,33]. This developmental trajectory is sensitive to environmental perturbations, including maternal stress [34].

Our finding of universal acceleration predominance—stronger in fetal than maternal heart rate and independent of sex or stress—may reflect fundamental developmental constraints on autonomic regulation. The predominance of heart rate accelerations over decelerations in healthy fetuses has been documented previously [35] and likely represents the balance between sympathetic and parasympathetic influences during normal third-trimester development.

The asymmetric coupling we observed, where maternal decelerations exert a stronger influence on fetal dynamics than maternal accelerations, may relate to the physiological salience of bradycardic events. Maternal heart rate decelerations could signal states requiring heightened maternal–fetal coordination, perhaps related to maternal respiratory patterns, as suggested by our previous work linking maternal breathing cycles to fetal heart rate responses [1]. The mechanical effects of diaphragm excursion on uterine pressure, combined with associated changes in maternal oxygenation and autonomic balance, may create particularly potent coupling conditions.

#### 4.3.3. Maternal–Fetal Physiological Communication Pathways

Multiple pathways can mediate maternal–fetal coupling. Direct mechanical transmission through uterine wall movement and amniotic fluid dynamics can influence fetal heart rate [36]. Shared placental circulation creates metabolic coupling, with maternal blood gas changes rapidly affecting fetal oxygenation [37]. Maternal autonomic fluctuations can alter uterine blood flow, indirectly influencing fetal cardiovascular regulation [38].

Our distinction between stress-sensitive temporal coupling and stress-invariant state-dependent coupling may reflect different communication pathways. The state-dependent coupling—robust across stress conditions and demographic variation—could represent direct mechanical or circulatory coupling that remains constant. The temporal coupling—modulated by stress and showing sex-specific patterns—might involve more complex autonomic and hormonal pathways susceptible to maternal stress effects.

This interpretation aligns with the “fetal stress memory” concept proposed in our previous work [1], in which stressed fetuses exhibited altered responses, suggesting persistent programming effects. The information-theoretical framework reveals that this programming may specifically affect temporal prediction dynamics while preserving fundamental state-dependent coordination mechanisms.

### 4.4. Sex Differences in Fetal Stress Responses: Exploratory Observations

Sex differences in prenatal development and stress vulnerability are increasingly recognized [39,40]. Male fetuses typically grow faster but may be more vulnerable to adverse prenatal conditions, while female fetuses show more adaptive physiological responses to environmental challenges [41,42].

Our exploratory sex-stratified analyses revealed striking patterns, though none survived correction for multiple comparisons and all require independent replication. Female fetuses showed numerous transfer entropy associations with maternal cortisol and neurodevelopmental outcomes that were entirely absent in males. Male fetuses instead showed entropy rate and sample entropy associations with motor development, particularly in the control subgroup, which disappeared under maternal stress conditions.

These tentative patterns align with the hypothesis that female fetuses may exhibit more pronounced maternal–fetal physiological coupling, potentially as an adaptive mechanism for monitoring maternal stress [43]. Male fetuses may rely more on signal complexity measures related to their own autonomic maturation, with maternal stress disrupting these developmental trajectories.

The robust sex-by-stress interaction in transfer entropy from our mixed linear model analysis provides the only statistically reliable evidence for sex-differentiated coupling mechanisms in our dataset. This finding warrants mechanistic investigation in adequately powered studies designed specifically to test sex-specific hypotheses.

Several biological mechanisms could underlie sexually dimorphic stress responses. Differential placental function by fetal sex has been documented, with female placentas showing more adaptive responses to maternal stress through altered gene expression and metabolic profiles [42,44]. Sex steroid hormones, present even in fetal life, can modulate HPA axis and autonomic nervous system development differently in males and females [40]. The timing of autonomic nervous system maturation differs between sexes, potentially creating windows of differential vulnerability [45].

### 4.5. Maternal–Fetal Coupling: Mechanisms, Quantification and Clinical Implications

#### 4.5.1. Why “Coupling”? Defining Physiological Interdependence

Before interpreting the asymmetric patterns we observed, it is essential to clarify what we mean by “coupling” and why this term appropriately describes our findings.

In physiological systems, coupling refers to the condition where two systems influence each other such that the state of one system systematically affects the dynamics of the other [46]. Coupled systems are not operating independently; rather, the state of one system constrains the accessible states of the other. This concept extends beyond mere statistical correlation to imply mechanistic (causal) physiological interdependence.

Our findings meet the criteria for true physiological coupling through several lines of evidence.

**Statistical dependency and state constraint:**

Fetal HR complexity is not independent of mHR state. When mHR decelerates, fHR complexity systematically changes (β=−0.123,p=0.012), demonstrating that maternal physiological state constrains the fetal state space. Fetal HR dynamics become more predictable and regular during mHR decelerations, indicating fewer accessible dynamical states, a hallmark of coupling in complex systems [47].

**Bidirectional asymmetric influence:**

We observe coupling in both directions—maternal states influence fetal dynamics (coupling strength 60%) and fetal states influence maternal dynamics (coupling strength 40%)—confirming true interdependence rather than unidirectional causation. The asymmetry in coupling strength (maternal→fetal stronger than fetal→maternal) indicates hierarchical physiological organization while preserving bidirectionality.

**Temporal coordination:**

The coupling effects occur during the physiological events (maternal/fetal HR decelerations), not at random times. Transfer entropy analyses reveal directional information flow with temporal lag, and our previous pPRSA work [1] demonstrated phase-synchronized fetal responses to maternal events. This temporal specificity distinguishes coupling from coincidental co-occurrence.

With this conceptual foundation established, we can now examine the quantitative strength and mechanistic basis of this coupling.

#### 4.5.2. Quantifying Coupling Strength: The 60% Ratio

The 60% coupling strength derives from the ratio of MLM beta coefficients: the maternal deceleration effect (β=−0.123) divided by the no-conditioning baseline (β=+0.206). This quantifies how strongly maternal bradycardic states constrain fetal HR complexity relative to the available dynamic range. This coupling is stress-invariant and represents a universal maternal–fetal coordination mechanism. See Supplementary Discussion, Section S3.1, for the complete mathematical derivation and the reconciliation with the stress-sensitive FSI [1].

### 4.6. Methodological Advances: The Three-Layer Conditioning Framework

Our conditioning framework systematically dissects univariate properties, self-conditioned properties, and cross-conditioned properties, isolating true bivariate coupling from coincidental state matching. The 60% coupling strength during maternal decelerations demonstrates genuine physiological constraint rather than mere correlation. See Supplementary Discussion, Section S3.2, for technical details.

### 4.7. From Information Metrics to Physiological Meaning

A central challenge in applying information-theoretical measures to physiological data is interpreting what changes in these metrics mean biologically. We address this for each of our principal findings.

**What does reduced fetal entropy during maternal decelerations mean?** Entropy rate quantifies the rate at which new, unpredictable information appears in a signal. When fetal entropy rate drops by 60% during maternal deceleration events, this indicates that fetal heart rate dynamics become substantially more predictable and constrained; the fetus is, in effect, “tracking” the maternal cardiovascular state rather than exhibiting independent autonomic variability. Physiologically, this likely reflects engagement of shared regulatory mechanisms: maternal vagal activation during bradycardic episodes may alter uterine hemodynamics and fetal oxygenation, compelling fetal cardiovascular regulation into a more constrained operating regime [36,38]. Importantly, this coupling is conserved across stressed and control pregnancies, suggesting it represents a fundamental feature of healthy maternal–fetal physiology, analogous to the tight cardiorespiratory coupling observed in healthy adults during sleep, where reduced autonomic variability reflects coordinated rather than impaired regulation [48].

**What does stress-sensitive transfer entropy imply for fetal development?** Transfer entropy quantifies directional information flow, specifically, the degree to which the maternal heart rate history improves prediction of fetal heart rate beyond the fetus’s own signal history. The stress modulation of this metric (*p* = 0.026) and the sex-by-stress interaction (*p* = 0.009) suggest that prenatal stress alters the temporal dynamics of how maternal cardiovascular information is transmitted to the fetus. This is consistent with the broader developmental programming literature, in which altered fetal autonomic responsiveness to maternal physiological cues has been linked to later cardiometabolic risk [27,28] and neurobehavioral outcomes [32,34]. In animal models, prenatal stress exposure produces lasting alterations in offspring heart rate variability and baroreflex sensitivity [49], providing a mechanistic link between altered information flow in utero and postnatal cardiovascular dysregulation.

**What does the null neurodevelopmental result mean?** None of our 144 entropy–neurodevelopment correlations survived FDR correction, and this should not be dismissed as merely a power limitation. It may reflect the genuine complexity of the causal chain: prenatal coupling metrics at a single third-trimester time point capture one snapshot of a dynamic developmental process, and their relationship to 24-month Bayley outcomes is mediated by birth events, postnatal environment, and continued maturation. The exploratory patterns we observed—TE associations with stress physiology, ER with motor development—generate testable hypotheses about domain-specific developmental pathways, but establishing these links requires longitudinal designs with repeated prenatal assessments and larger samples.

### 4.8. Clinical and Physiological Implications

**Extending biomarker development:**

The Fetal Stress Index [1] demonstrated clinical potential for identifying fetuses exposed to maternal stress. The information-theoretical framework developed here suggests that multiple complementary biomarkers may be needed. Transfer entropy may capture stress-sensitive temporal coupling, while conditioned entropy measures fundamental coordination mechanisms. A multimodal approach combining these measures could improve both sensitivity and mechanistic understanding.

**Neurodevelopmental prediction:**

While our exploratory entropy–neurodevelopment associations did not survive correction for multiple comparisons, the tentative patterns suggesting domain-specific relationships—transfer entropy with stress physiology, entropy rate with motor development, sample entropy with language—warrant investigation in larger samples. If replicated, epoch-specific entropy features computed during acceleration and deceleration events may prove more informative than full-recording features, consistent with the state-dependent nature of coupling we observed.

**Fundamental coupling mechanisms:**

The stress-invariant state-dependent coupling we identified may represent a universal maternal–fetal coordination mechanism, conserved across demographic variation and robust to maternal stress effects. This fundamental coupling could serve as a reference against which pathological conditions (severe stress, placental dysfunction, fetal growth restriction) could be evaluated. Deviations from normal state-dependent coupling might indicate compromised maternal–fetal communication requiring clinical attention.

**Sex-specific vulnerabilities:**

The exploratory observation of opposite coupling patterns in male and female fetuses, if replicated, could have implications for sex-specific clinical monitoring. Female fetuses might require different assessment approaches than male fetuses, with coupling measures potentially offering complementary information to traditional fetal monitoring parameters.

### 4.9. Limitations and Future Directions

**Sample size constraints:** With 118 participants contributing 50 entropy features, our study was adequately powered for mixed linear model analyses with focused predictors but severely underpowered for multivariate modeling. The exploratory correlation analyses revealed numerous associations that did not survive correction for multiple comparisons. These null FDR results do not prove the absence of relationships; they reflect insufficient power for the large number of statistical tests performed. Future studies should employ sample sizes of n > 500 to enable robust multivariate analysis and adequately powered subgroup analyses while maintaining control for multiple comparisons. Furthermore, given the severe multicollinearity among entropy features (91–94% with VIF > 10), hierarchical clustering of correlated metrics before statistical testing could reduce the effective number of comparisons, potentially allowing meaningful associations to survive multiple comparison correction. This data-driven dimensionality reduction approach may prove more appropriate than the standard Benjamini–Hochberg correction, which assumes several independent tests that substantially exceed the true degrees of freedom in our highly correlated feature space.

**Neurodevelopmental follow-up:** Bayley assessments were available for approximately 55% of the cohort, with sample sizes for specific developmental domains ranging from 30 to 66 participants. While sufficient for exploratory correlation analysis, these samples were too small for definitive conclusions about entropy–development relationships. Complete follow-up in larger cohorts is essential for validating potential neurodevelopmental associations.

**Stress measurement:** Our binary stress classification (stressed vs. control) based on maternal report may not capture the full stress continuum or distinguish chronic from acute stress effects. Future work should incorporate: continuous stress measures enabling dose-response analyses; physiological stress biomarkers beyond cortisol (inflammatory markers, autonomic function); longitudinal stress trajectories across pregnancy; and assessment of specific stress types (anxiety vs. depression vs. life events).

**Relation with maternal respiratory phase:** Respiratory data were not directly collected in the current study; our discussion of respiratory–cardiovascular coupling mechanisms remains interpretive. However, ECG-derived respiration (EDR) based on respiratory sinus arrhythmia (RSA) can be extracted retrospectively from the existing RR interval time series: since RSA is encoded in beat-to-beat timing rather than ECG morphology, it is unaffected by the abdominal electrode placement or the SAVER source-separation algorithm used for maternal–fetal ECG deconvolution. Conditioning the transfer entropy analysis on maternal respiratory phase estimated from RSA would directly test whether the observed coupling is respiratory-mediated, and is planned as a follow-up study, though validation against direct respiratory measurements in pregnant women would first be required.

**Sample entropy vs. entropy rate:** Despite adequate data quality for sample entropy (99.5% non-zero observations in conditioned windows, 2348 total observations, see Supplementary Table S7 for details), SE did not detect the bivariate coupling signatures identified by ER. This confirms that the distinction is not methodological (data sparsity) but reflects a genuine difference in what these metrics capture: SE measures signal regularity and complexity, while ER captures the rate of new information generation. The cross-signal coupling from maternal decelerations to fetal HR complexity is a temporal-predictive phenomenon best captured by ER. Sample entropy, in contrast, is sensitive to within-signal state changes (e.g., maternal HR during maternal accelerations, β=−0.223,p<0.001) but not to cross-signal coupling.

**Mechanistic validation:** While our information-theoretical analyses reveal coupling patterns, they do not directly identify the physiological mechanisms mediating maternal–fetal communication. Future work should combine entropy analyses with: simultaneous measurement of potential mediators (uterine blood flow, amniotic pressure, maternal oxygenation); experimental manipulations in animal models where mechanistic pathways can be directly tested; computational modeling to determine which physiological pathways can account for observed coupling patterns; and integration with placental function measures to assess the role of placental health in coupling strength.

**Replication imperative:** The sex-stratified patterns and entropy–outcome associations reported here are exploratory, hypothesis-generating findings that did not survive correction for multiple comparisons. Independent replication in adequately powered samples with pre-registered analysis plans is essential before these patterns inform biological interpretation or clinical application. The robust findings—mixed linear model results for acceleration/deceleration asymmetry, conditioned entropy coupling, and sex-by-stress interactions in transfer entropy—provide the foundation for reliable biological inference and should be the focus of mechanistic follow-up.

## 5. Conclusions

This study provides the first comprehensive characterization of time-scale-dependent information flow between maternal and fetal heart rates during the third trimester, revealing fundamental insights into prenatal cardiac coupling mechanisms. Our findings demonstrate that maternal–fetal heart rate interactions operate through dual mechanisms: a robust, stress-invariant state-dependent synchronization and a more labile, stress-sensitive temporal information transfer pathway.

The discovery that maternal heart rate decelerations influence fetal entropy rate at approximately 60% of the mother’s self-regulatory coupling strength represents a novel quantitative benchmark for healthy maternal–fetal physiological integration. This “60% rule” persists across stress conditions, suggesting it reflects a fundamental biological constraint rather than an adaptive response.

Critically, we identified a sex-by-stress interaction in transfer entropy measures, with male fetuses from stressed pregnancies showing altered information transfer patterns. This finding aligns with known sex differences in prenatal stress vulnerability and provides a potential mechanistic explanation for differential developmental programming outcomes.

The universal predominance of acceleration-associated coupling, independent of stress exposure, indicates that cardio-acceleration represents the primary mode of maternal–fetal heart rate synchronization during healthy third-trimester pregnancy. Furthermore, our demonstration that 4 Hz sampling rates capture the essential physiological dynamics validates the clinical applicability of standard fetal monitoring equipment for assessing maternal–fetal coupling.

Future research should extend these methods to high-risk pregnancies and investigate whether transfer entropy measures can serve as early biomarkers for adverse developmental outcomes.

## Figures and Tables

**Table 2:** Baseline characteristics by fHR analysis inclusion status.

Variable	All (N = 165)	Included (n = 118)	Excluded (n = 46)	*p*-Value
**Sociodemographic Characteristics**				
Maternal age (years)	33.1 ± 4.4	33.0 ± 4.6	33.4 ± 4.0	0.557
University degree	112 [67.9%]	79 [66.4%]	33 [71.7%]	0.635
High income (>5000€/month)	78 [47.3%]	49 [41.2%]	29 [63.0%]	0.019 *
Married	123 [75.0%]	88 [74.6%]	35 [76.1%]	1.000
Primipara	89 [53.9%]	59 [49.6%]	30 [65.2%]	0.102
Unplanned pregnancy	35 [21.3%]	29 [24.6%]	6 [13.0%]	0.159
Smoking during pregnancy	8 [4.8%]	6 [5.0%]	2 [4.3%]	1.000
Autoimmune disease	19 [11.5%]	16 [13.4%]	3 [6.5%]	0.282
**Prenatal Exposure Measures**				
PSS-10 score	17.0 (13.0)	18.5 (12.0)	11.5 (15.8)	0.145
PDQ score	11.0 (11.0)	11.0 (10.0)	9.0 (9.8)	0.265
Hair cortisol (pg/mg)	92.0 (109.0)	78.0 (95.0)	117.0 (90.5)	0.034 *
Stress group	79 [47.9%]	59 [49.6%]	20 [43.5%]	0.596
**Pregnancy Outcomes**				
Any complications	19 [11.5%]	14 [11.8%]	5 [10.9%]	1.000
Gestational age at birth (weeks)	39.7 (1.7)	39.7 (1.9)	39.6 (1.6)	0.705
Birth weight (grams)	3510.5 ± 430.5	3532.6 ± 406.6	3454.2 ± 486.2	0.297
Child sex (male)	71 [43.6%]	49 [41.9%]	22 [47.8%]	0.599

Notes. For continuous variables, results are shown as mean ± standard deviation for normally distributed data or median (interquartile range) for non-normally distributed data. For dichotomous variables, results are reported as the total number of positive cases [proportion]. Any complications were defined as the presence of at least one of the following: hypertensive disorder, preeclampsia, eclampsia, HELLP syndrome, gestational diabetes, or intrauterine growth restriction. *: *p*-value < 0.05.

**Table 3:** Sample entropy associations with neurodevelopmental outcomes (exploratory).

Feature	Outcome	r	*p*	q (FDR)	Method	n
SE fetus (fHR accel)	Language receptive	+0.290	0.026	0.62	Spearman	59
SE mother (fHR accel)	Language receptive	+0.290	0.026	0.62	Spearman	59

Note. SE = sample entropy; ER = entropy rate; mHR = maternal heart rate; fHR = fetal heart rate; accel = acceleration epochs; decel = deceleration epochs. Both q-values = 0.62, indicating failure to survive FDR correction. No ER features reached *p* < 0.05 in the overall sample. Previously reported ER/SE–Bayley associations from stratified analyses (by sex or stress group) are not included in this table as they represent post hoc subgroup findings with even greater multiple-comparison burden.

**Table 6:** Transfer entropy association with neurodevelopment (exploratory).

Feature	Outcome	r	*p*	q (FDR)	n
Max TE, conditioned on fHR accel.	Language Expressive	−0.277	0.036	0.73	58

Note. All correlations were computed using the Spearman method. TE = transfer entropy; fHR = fetal heart rate. q = 0.73 indicates failure to survive FDR correction. No other TE–Bayley associations reached p<0.05.

## Data Availability

The data presented in this study are available on request from the corresponding author. The data are not publicly available due to privacy and ethical restrictions concerning human participants. Code is available at https://github.com/martinfrasch/felicity1_te (accessed on 5 May 2026).

## Supplementary Materials

The following supporting information can be downloaded at: https://www.mdpi.com/article/10.3390/biology15100749/s1. Refs. [50,51,52,53,54] are cited in Supplementary Materials.
