# Supplementary material for: Measuring the Time-Scale-Dependent Information Flow Between Maternal and Fetal Heartbeats During the Third Trimester: Impact of Fetal Sex and Maternal Chronic Stress
Source: Biology (Basel). 2026 May 9;15(10):749. doi: 10.3390/biology15100749 (PMC13203766; doi:10.3390/biology15100749)
Supplement: Supplementary file 1 [file biology-15-00749-s001.zip › biology-4161529-supplementary.pdf]

biology

MDPI

Article

Supplementary Materials: Measuring the time-scale-dependent information flow between maternal and fetal heartbeats during the third trimester: impact of fetal sex and maternal chronic stress

Nicolas B. Garnier [1]*, Maria S. Molinet [2], Marta C. Antonelli [2,3], Silvia M. Lobmaier [2] and Martin G. Frasch [4,5]*

1 CNRS, ENS de Lyon, LPENSL, UMR5672, 69342, Lyon cedex 07, France; nicolas.garnier@ens-lyon.fr
2 Department of Obstetrics and Gynecology, TUM University Hospital of Technical University of Munich, TUM School of Medicine, Technical University of Munich, Germany; silvia.lobmaier@tum.de
3 Instituto de Biología Celular y Neurociencia "Prof. E. De Robertis", Facultad de Medicina, Universidad de Buenos Aires, Buenos Aires, Argentina; mca@fmed.uba.ar
4 Dept. of Obstetrics and Gynecology, School of Medicine, University of Washington, Seattle, WA, USA
5 Institute on Human Development and Disability, School of Medicine, University of Washington, Seattle, WA, USA; mfrasch@uw.edu
* Correspondence: nicolas.garnier@ens-lyon.fr and mfrasch@uw.edu

Abstract

This document provides supplementary materials for the main manuscript, including detailed mathematical definitions, model specifications, additional results, and technical discussion. Content is organized to ensure full reproducibility while keeping the main text accessible to a biology-oriented readership.

Contents

S1. Supplementary Methods 3
S1.1. Low-pass Filtering Details 3
S1.2. Mathematical Definitions of HR Decelerations and Accelerations 4
S1.3. Entropy Rate Formulas 5
S1.4. Transfer Entropy Formulas 5
S1.5. Conditioning Framework Details 6
S1.6. Mixed Linear Model Specifications 7
S1.7. Sensitivity Analysis Details 7
S1.8. Net Transfer Entropy Significance Testing (Table 4) 7
S1.9. Multivariate Modeling Methods 8

S2. Supplementary Results 8
S2.1. Time-scale Identification Details 8
S2.2. Entropy Progression Reveals Coupling Hierarchy 9
S2.3. Multivariate Modeling Results 9
S2.3.1. Multicollinearity Assessment 9
S2.3.2. Model Performance 9
S2.3.3. Feature Selection Patterns 10
S2.3.4. PLS Loading Analysis 10
S2.3.5. Parsimonious Forward Selection Results 10
S2.3.6. Integration and Interpretation 11
S2.4. Dependence of TE on HR Sampling Rate 11

Check for updates

Received:
Revised:
Accepted:
Published:

Copyright: © 2025 by the authors. Licensee MDPI, Basel, Switzerland. This article is an open access article distributed under the terms and conditions of the Creative Commons Attribution (CC BY) license.

 https://doi.org/10.3390/biology1010000

S3. Supplementary Discussion **12**
S3.1. Quantifying Coupling Strength: Mathematical Details 12
S3.1.1. Reconciling the 60% Coupling with Stress-Sensitive FSI 12
S3.2. The Three-Layer Conditioning Framework 13

S4. Supplementary Box: Understanding Beta Coefficient Derivation **14**

S5. References **14**

S6. Supplementary Tables **16**

S7. Supplementary Figures **18**

https://doi.org/10.3390/biology1010000

S1. Supplementary Methods

S1.1. Low-pass Filtering Details

The raw heart rate X signal is derived from the successive time positions of the R-peaks of the ECG signal, given by SAVEr with a resolution f_{ECG}, as follows. This raw HR signal is a step-wise function of time sampled at f_{ECG}: its value is constant between two consecutive R peaks. Indeed, as time passes, its value changes when, and only when, a new R peak occurs: it is then possible to compute the size of the RR-interval that just ended when the new R peak occurs. A new value of the raw heart rate can then be deduced, which is constant until the next R-peak occurs. To have a continuously evolving HR signal sampled at a given frequency f_s — possibly much larger than the typical frequency associated with RR-intervals or heart rate —, we first low-pass filter the raw HR signal by using a local averaging over a time interval corresponding exactly to the timescale τ we are willing to study.

$$X_\tau(t) = \frac{1}{\tau}\int_{t'=t-\tau}^{t} X(t')dt' \tag{S1}$$

$$= \frac{1}{\tau f_{\mathrm{ECG}}} \sum_{k=k_{t-\tau}+1}^{k_t} X_k\,, \tag{S2}$$

where we have noted $k_t = t f_{\mathrm{ECG}}$ the index of the point at time t in a signal sampled at f_{ECG}. The first eq.(S1) is formal and relates to a time-continuous signal while the second eq.(S2) is the one used in practice. This procedure is depicted in Fig. S1.

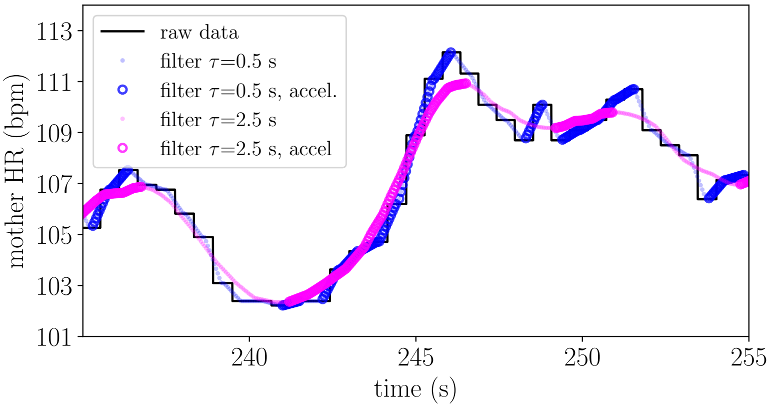

Figure S1. Example of mother HR (mHR) data. The raw mHR sampled at 1kHz is a stepwise function of time (black). We low-pass filter the raw mHR signal using a low-pass cutoff frequency $1/\tau$, where the time-scale τ represents the scale at which we will further analyze the information contents of the heart rates. Low-pass-filtered signals (sampled at 1kHz, same as for the raw HR) are then downsampled at f_s=20Hz, independent of the time-scale of interest. Two examples are represented: one for τ=0.5s (blue) and one for τ=2s (magenta). Larger circles indicate times when the filtered mHR signal is increasing, which we define as *accelerations*. Conversely, little dots correspond to *decelerations*.

Filtering removes noise and information at frequencies higher than the cutoff frequency $1/\tau$ which corresponds to the timescale τ we are studying. For example, the SAVER algorithm is upsampling to 1000 Hz the aECG data from AN24 recorded at 900 Hz; this interpolation introduces some higher frequency noise, which is removed by the filtering. In the following, we vary τ in the range [0.5s-20s] to explore the distribution of information in the heart rates. The lowest value $\tau = 0.5$s in this range corresponds to the typical time interval between two consecutive R-peaks: examining time-scales lower than 0.5s is thus

illusory, as there is no information in the raw HR signal at these scales (see the black curve in Figure S1). The larger value $\tau = 20$s is sufficient to retain all the interesting behavior of the interactions we are looking for [1].

After filtering, the data X_τ is down-sampled at a fixed sampling frequency f_s which we set to 20Hz, unless noted otherwise in Section S2.4 where we specifically study the effect of the sampling frequency.

S1.2. Mathematical Definitions of HR Decelerations and Accelerations

We define HR accelerations and decelerations [2] at a given time-scale τ as follows. For a given HR signal X, we first low-pass filter this signal as described above using the time-scale τ, and then examine the sign of the time-derivative of the filtered signal. We define accelerations $\mathcal{A}_\tau$ as the set of times (epochs) where the time-derivative of the filtered signal X_τ is positive:

$$t \in \mathcal{A}_\tau \Leftrightarrow \frac{dX_\tau(t)}{dt} > \mu, \qquad \text{with} \quad \mu = 0 \tag{S3}$$

Respectively, we define decelerations $\mathcal{D}_\tau$ as the set of times where the time-derivative is negative:

$$t \in \mathcal{D}_\tau \Leftrightarrow \frac{dX_\tau(t)}{dt} < -\mu, \qquad \text{with} \quad \mu = 0 \tag{S4}$$

Because the value of the time-derivative depends on the time-scale of the filter, so does the partitioning of epochs in accelerations and decelerations, as can be seen in Fig. S1. At a given timescale τ, accelerations, resp. decelerations occur in sets of consecutive times (epochs), the duration of which is typically larger than τ. As a consequence, for smaller time-scales τ we expect a larger number of distinct — *i.e.*, well-separated in time — accelerations, resp. decelerations, and for larger time-scales we expect a smaller number of distinct accelerations, resp. decelerations, while each is expected to be "longer" in that it should contain more points.

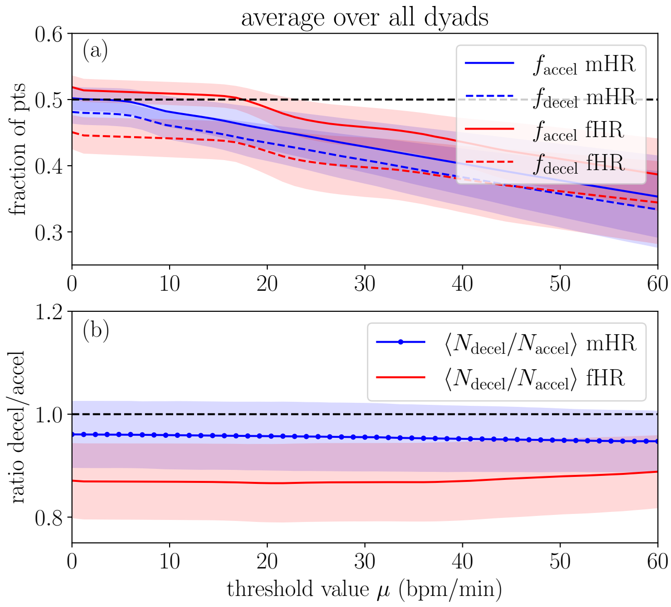

Figure S2. Effect of an increasing threshold value μ (expressed in slope units bpm/minute, *i.e.*, beat per squared minutes) on (a): the fraction of points from mHR (blue) and fHR (red) that are detected in accelerations $\mathcal{A}_\tau$ (plain lines) or decelerations $\mathcal{D}_\tau$ (dotted lines) epochs; and (b): the ratio of decelerations to accelerations, which is always constant and below 1. Dotted black lines indicate values 50% in (a) and 1 in (b). The time-scale was fixed to $\tau = 1$s.

https://doi.org/10.3390/biology1010000

Our method can be tuned by requiring that the absolute value of the time derivative exceed a finite threshold $\mu \neq 0$. Still, our explorations showed that increasing the threshold severely reduces the number of points N_{accel}, resp. N_{decel}, in acceleration, resp. deceleration, epochs, see Figure S2(a). This is expected, as increasing τ smoothens the HR signal more, thus reducing its dynamics: the standard deviation of X_τ, and hence of its time-derivative, is typically $\sqrt{\tau}$ times smaller than the standard deviation of X. It can nevertheless be noted that the ratio $N_{\text{decel}}/N_{\text{accel}}$ of decelerations to accelerations remains roughly constant and below 1 (see Figure S2(b)), which suggests that increasing the threshold only alters the number of points available for our analysis, and should not impact our findings. In this article, we chose a threshold $\mu = 0$ to ensure sufficient points for accelerations and decelerations.

S1.3. Entropy Rate Formulas

To get some insight into the information content of the conditioned signals, we measured their entropy rate [3–5] over the time-scale τ:

$$h(\tau) = H(X_\tau(t), X_\tau(t-\tau)) - H(X_\tau(t-\tau)) \tag{S5}$$

$$= H^{(2)}(X_\tau) - H(X_\tau) \tag{S6}$$

where $H(X_\tau)$ is the Shannon entropy of the filtered signal X_τ and $H^{(2)}(X_\tau)$ is the Shannon entropy of the bi-variate filtered signal $(X_\tau(t), X_\tau(t-\tau))$, obtained by time-embedding the filtered signal X_τ over the time-scale τ. Unless noted otherwise, this study uses filtered data sampled at f_s =20Hz.

We computed the entropy rate for a set of time-scales $\tau \in [0.05; 20]$ seconds. Because we are interested in time-scale range [0.5; 2.5]s, we compute the maximal value $h^{\max}$ of the entropy rate in that range, as well as its mean value h^{AUC} in the same range, which up to a factor 2s represents the area under the curve (AUC) of $h(\tau)$ in the range [0.5; 2.5]s.

S1.4. Transfer Entropy Formulas

To explore the respective influences of mother and foetal heart rates, we compute the transfer entropy between the filtered heart rates of the mother and her foetus. Transfer entropy was introduced by Schreiber [6] and has since gained widespread popularity to study information exchange between two signals in a wide range of systems, and in particular maternal and fetal heart rates: first using 1-minute long ECG data [1], or using fetal magnetocardiography data [7], see [8] for an exhaustive review. In particular, maternal to fetal coupling was shown to increase from early to mid and then to late gestation while fetal to maternal coupling do not change significantly [1], albeit non-stationary effects like fetal movements may blur this observation [7].

For two signals $X(t)$ and $Y(t)$ and a positive time lag τ', transfer entropy $\text{TE}^{(\tau')}(X(t) \rightarrow Y(t))$ expresses the amount of shared information between $X(t)$ and $Y(t+\tau')$ that is not contained in $Y(t)$. In other words, it quantifies how much information in the future of Y at time $t+\tau'$ is also present in the present of X at time t, while not being present in Y itself at time t. It is thus interpreted as the amount of information that flows from X to Y on the time-scale τ'.

Noting M_τ and F_τ the maternal and the fetal heart rates filtered at the time-scale τ, we naturally set the time-lag $\tau' = \tau$ to define

$$\text{TE}_{m\rightarrow f}(\tau) = \text{TE}^{(\tau)}(M_\tau(t) \rightarrow F_\tau(t)) \tag{S7}$$

$$\text{TE}_{f\rightarrow m}(\tau) = \text{TE}^{(\tau)}(F_\tau(t) \rightarrow M_\tau(t)) \tag{S8}$$

We also define the "net" TE from mother to fetus as the difference:

$$\mathrm{TE}(\tau) = \mathrm{TE}_{m \to f}(\tau) - \mathrm{TE}_{f \to m}(\tau)\,, \tag{S9}$$

which is positive when more information flows from the mother to her foetus than information flows from the foetus to the mother, and negative otherwise.

The TE is measured using a nearest neighbors estimator with $k = 5$ neighbors [9] and using 4000 points. We estimate the bias of this estimator by computing the TE with surrogate data, as follows. For each set of signals (X, Y), we construct 10 sets of surrogate signals (X_s, Y_s) by shuffling the original signals, which destroys the complete time dependencies between X and Y. We then measure $\mathrm{TE}(X_s \to Y)$ and $\mathrm{TE}(Y_s \to X)$. The values we obtain are very small (of the order 10^{-3}). We average these values over the set of 10 surrogates and subtract this estimate of the bias from the $\mathrm{TE}(X \to Y)$ and $\mathrm{TE}(Y \to X)$, respectively.

Because a dataset contains much more than 4000 points in time, we compute the TE on 10 different subsets of 4000 points, randomly picked in the dataset. This allows us to estimate the standard deviation of the TE estimator, which we use to represent error bars in the figures. For a given subject, these error bars are small.

We computed the entropy rate for a set of time-scales $\tau \in [0.05; 20]$ seconds, with a step $1/f_s = 0.05$s. The typical evolution of the net TE averaged over the cohort as a function of the time-scale τ can be seen in Figure S3. Because we are interested in time-scale range $[0.5; 2.5]$s, we compute the maximal value $\mathrm{TE}^{\max}$ of the TE in that range, as well as the mean value $\mathrm{TE}^{\mathrm{AUC}}$ of the TE within that range, which up to a factor 2s represents the area under the curve (AUC) of $\mathrm{TE}(\tau)$ in that range.

S1.5. Conditioning Framework Details

We computed univariate entropy metrics (ER and SE) at three levels of analysis, using the conditioning procedure described in the main text, in order to characterize both univariate signal properties and bivariate maternal-fetal coupling:

1. Univariate baseline: **Entropy rate (ER)/Sample Entropy (SE)** computed on the complete fetal or maternal HR time series without conditioning (no_conditioning), capturing baseline complexity of each signal independently.
2. Self-conditioned: ER/SE of a signal during its own detected events (e.g., fetal HR during fetal accelerations), capturing state-dependent complexity within the same signal.
3. Cross-conditioned bivariate: ER/SE of one signal (e.g., fetal HR) computed specifically during events detected in the other signal (e.g., maternal accelerations or decelerations). This framework inherently captures maternal-fetal coupling: if fetal entropy differs when conditioned on maternal events versus no conditioning, this reveals that mHR state modulates fHR complexity - a signature of physiological interdependence.

We also computed transfer entropy (TE), which is already bivariate, using either the full fHR and mHR time series, or using conditioning.

So in this framework, each quantity was computed under five conditioning paradigms: (1) full recording, (2) fetal HR acceleration epochs, (3) fetal HR deceleration epochs, (4) maternal HR acceleration epochs, and (5) maternal HR deceleration epochs. This yielded

- 20 ER features: max/mean × fetus/mother × {full, fHR_accel, fHR_decel, mHR_accel, mHR_decel}
- 20 SE features: same structure as ER
- 10 TE features: max/mean × {full, fHR_accel, fHR_decel, mHR_accel, mHR_decel}
- Total: 50 entropy-based features

S1.6. Mixed Linear Model Specifications

To properly account for repeated measures within subjects and enable interaction testing, we employed mixed linear models (MLMs) with restricted maximum likelihood (REML) estimation for three separate analyses:

Model 1 - Acceleration/Deceleration Ratios:

$$\texttt{Fraction} \sim \texttt{Sex} \times \texttt{Stress} \times \texttt{HR\_Source} \times \texttt{Event\_Type} + (1|\texttt{Patient\_ID})$$

Data structure: 472 observations (118 patients × 4 measurements each: mHR_accel, mHR_decel, fHR_accel, fHR_decel). Random intercept accounts for patient-level correlation.

Model 2 - Entropy Rate with Conditioning:

$$\texttt{Value} \sim \texttt{Sex} \times \texttt{Stress} \times \texttt{Metric} \times \texttt{HR\_Source} \times \texttt{Conditioning} + (1|\texttt{Patient\_ID})$$

Data structure: 1,006 observations (average 8.5 per patient). Conditioning levels included: none (univariate baseline), mother_accel, mother_decel, fetus_accel, and fetus_decel (cross-conditioned bivariate measures). Selected 2-way interactions included based on theoretical relevance.

Model 3 - Sample Entropy with Conditioning:

$$\texttt{Value} \sim \texttt{Sex} \times \texttt{Stress} + \texttt{Metric} + \texttt{HR\_Source} \times \texttt{Conditioning} + (1|\texttt{Patient\_ID})$$

Sample entropy was computed using the same conditioning framework as the entropy rate. Recomputed sample entropy values yielded 99.5% non-zero observations across all conditioning types, enabling a full MLM analysis with the same conditioning structure as ER. Data structure: 2,348 observations (19.9 per patient, comparable to entropy rate).

Rationale for MLM: Each patient contributes multiple measurements, creating within-subject correlation. Independent t-tests would treat these as independent observations, leading to inflated effective sample size, underestimated standard errors, and artificially low p-values (pseudoreplication). Random intercepts for Patient_ID provide valid statistical inference for hierarchical data.

All models included two-way interactions between fixed effects. Statistical significance was assessed at $\alpha = 0.05$. Analyses were conducted in Python 3.9 using statsmodels 0.14.4.

S1.7. Sensitivity Analysis Details

To assess robustness to potential confounders, we repeated all MLM analyses with gestational age at birth, maternal age, and pre-gestational BMI as additional covariates. Across all models (entropy rate, sample entropy, and transfer entropy), no originally significant effect changed significance status, with maximum coefficient changes of 7% (ER), 6% (SE), and 18% (TE). These results confirm that the reported associations are robust to adjustment for these demographic covariates.

S1.8. Net Transfer Entropy Significance Testing (Table 4)

The p-values reported in Table 4 of the main text quantify the probability of observing a zero or negative net transfer entropy under a Gaussian assumption. For a set of net TE values $\{x_i\}$ across the cohort, we compute the sample mean $\bar{x}$ and variance σ^2, then evaluate:

$$p = \int_{-\infty}^{0} \frac{1}{\sqrt{2\pi\sigma^2}} \exp\left(-\frac{(x-\bar{x})^2}{2\sigma^2}\right) dx \quad \text{(S10)}$$

This represents the estimated fraction of the population for which the net information flow would be directed from fetus to mother (i.e., reversed), under the assumption that inter-individual variability in net TE follows a Gaussian distribution. A small p indicates that most mother-fetus pairs exhibit net information flow from mother to fetus, with little overlap with zero. This differs from a standard one-sample t-test, which tests whether the *population mean* differs from zero; the present method instead characterizes the *consistency* of the directional information flow across the cohort. One participant (FS-124) was excluded from this analysis due to an extreme outlier value (z-score = 23.7).

S1.9. Multivariate Modeling Methods

To assess whether entropy features jointly predict neurodevelopmental outcomes and whether maternal stress moderates these relationships, we applied multiple regularization and dimensionality reduction approaches to handle the high-dimensional feature space (50 predictors: 20 ER + 20 SE + 10 TE, plus stress and sex indicators).

Methods Applied:

1. **Elastic Net Regression**: Combined L1 (Lasso) and L2 (Ridge) penalties to handle multicollinearity and perform automatic feature selection. The mixing parameter α was optimized via 5-fold cross-validation.
2. **PCA + Ridge Regression**: Principal component analysis followed by Ridge regression to address multicollinearity through orthogonal transformation of features. Components explaining 95% cumulative variance were retained.
3. **Partial Least Squares (PLS) Regression**: Designed specifically for high-dimensional, multicollinear predictors. Identifies latent components that maximize covariance between features and outcomes.
4. **Random Forest Regression**: Non-parametric ensemble method capturing non-linear relationships and interactions without assumptions about feature distributions.
5. **Parsimonious Forward Selection**: Ordinary least squares regression with forward feature selection limited to a maximum of 3 features, appropriate for small sample sizes.

Pre-processing: All features were standardized (z-scored) before modeling. Cross-validation employed 5-fold CV where sample size permitted (reduced for outcomes with $n < 20$).

Multicollinearity Assessment: Variance Inflation Factor (VIF) was calculated using statsmodels to quantify multicollinearity among predictors. VIF > 10 indicates severe multicollinearity requiring regularization approaches.

Sample Size Considerations: The ratio of sample size n to number of predictors k (n/k ratio) should ideally exceed 10-20 for reliable multivariate inference. With neurodevelopmental outcomes available for $n = 30$–66 participants and 52 total predictors (50 features + stress + sex), the n/k ratios ranged from 0.6 to 1.2, indicating severe underpowering for standard regression approaches.

S2. Supplementary Results

S2.1. Time-scale Identification Details

We present in the main text (Figure 5) the evolution of the entropy rate with the time-scale τ, averaged over the cohort, for the maternal and fetal heart rate signals. Here we provide additional interpretation.

We observe that the entropy rate is roughly constant across all time-scales, except for time-scales below 0.5-1s. This is expected as there is no information in the HR signal

Table S1. Cross-validated R^2 by model and outcome

Outcome	Elastic Net	PCA+Ridge	PLS	Random Forest
Cognitive Composite	-0.10	-0.06	-0.79	-0.10
Language Composite	-0.42	-0.57	-1.23	-0.40
Motor Composite	-0.49	-0.52	-0.99	-0.43
Motor Fine Skills	-0.23	-0.01	+0.10	-0.19
Motor Gross Skills	-0.18	-0.04	-0.52	-0.15

between consecutive RR peaks, which are typically separated by 0.5s for fHR and 0.8s for mHR. The same behavior is observed for the Sample Entropy.

When no conditioning is used, the entropy rate is always greater than that of any counterpart with conditioning; this holds for both fHR and mHR signals. We observe that whether with or without conditioning, the fHR always has a higher entropy rate than the mHR, except when conditioning using the fHR accelerations. In this latter case only, fHR appears to have a lower entropy rate than mHR in the band [0.5-2.5] s.

S2.2. Entropy Progression Reveals Coupling Hierarchy

From higher entropy rate to lower entropy rate, we found the following ordering:

- no conditioning (univariate baseline): highest entropy
- fetal acceleration conditioning (reference): $\downarrow \beta = -0.082$ $(p = 0.054,$ trend$)$
- fetal deceleration conditioning: $\downarrow \beta = -0.123$ $(p = 0.012^*)$
- maternal deceleration conditioning: lowest entropy (strongest coupling)

The progressive entropy reduction from univariate (no conditioning) to cross-conditioned demonstrates:

1. Conditioning constrains signal complexity (reduces entropy);
2. Cross-conditioning on the OTHER signal's events reveals bivariate coupling;
3. Maternal deceleration events exert the strongest influence on fetal HR predictability. The lowest entropy indicates here the strongest coupling.

S2.3. Multivariate Modeling Results

Multivariate analysis examined whether entropy features jointly predict neurodevelopmental outcomes using regularized regression and dimensionality reduction approaches designed for high-dimensional data. However, severe sample-size limitations relative to the number of predictors (50 entropy features + stress and sex indicators) prevented reliable multivariate inference. The n/k ratio (sample size/predictors ratio) should ideally exceed 10-20 for reliable multivariate inference. The observed ratios of $\sim$1.2 indicate severe underpowering, approximately 10-fold below recommended thresholds.

S2.3.1. Multicollinearity Assessment

Variance Inflation Factor analysis revealed severe multicollinearity among entropy features. Across outcomes, 91–94% of features (48–50 of 52) had VIF > 10. Highest VIF ($>$100) was obtained with max TE and mean TE, not using any conditioning. These are mathematically related, explaining the extreme multicollinearity. This indicates severe redundancy that necessitates regularization approaches but also reflects fundamental mathematical relationships between features computed from the same time series.

S2.3.2. Model Performance

Cross-validated R^2 values for multivariate models are presented in Table S1. Negative CV-R^2 values indicate that models performed worse than a null model predicting the sam-

https://doi.org/10.3390/biology1010000

Table S2. Elastic Net selected features for cognitive performance composite

Feature	Coefficient
SE mother (on mHR accel)	+0.095
stress_binary	-0.094
max_TE (no conditioning)	-0.067
SE fetus (on mHR accel)	+0.053

Table S3. Random Forest top TE features for Bayley test

Outcome	Top TE Features	Importance Score
Cognitive	mean TE, no conditioning	0.065
Cognitive	max TE, no conditioning	0.061
Motor Composite	max TE, no conditioning	0.079
Motor Composite	max TE on mHR decel	0.071
Motor Fine Skills	max TE on mHR decel	0.092

ple mean, reflecting overfitting due to the unfavorable ratio of predictors to observations. Only PLS regression for Motor Fine Skills achieved a marginally positive CV-R^2 of 0.10, explaining 10% of the variance.

S2.3.3. Feature Selection Patterns

Elastic Net selected features are shown by way of an example for the cognitive composite (Table S2): TE features were selected by Elastic Net with moderate coefficients, though model performance remained poor (CV-R^2 = -0.10).

Random Forest Feature Importance (Top TE Features, Table S3): TE features consistently ranked among the top Random Forest predictors, particularly for motor outcomes, despite overall poor model performance.

S2.3.4. PLS Loading Analysis

Partial least squares regression identified latent components with the highest feature loadings (Table S4).

Pattern: TE features dominated PLS loadings for cognitive and motor outcomes, while ER features dominated for language outcomes, suggesting domain-specific relevance despite poor overall predictive performance.

S2.3.5. Parsimonious Forward Selection Results

Using a maximum of 3 features to optimize the n/k ratio:

Cognitive Composite: Selected: mean TE (no conditioning), SE mother (conditioned on mHR accel). R^2(adj) = 0.067. No significant predictors.

Table S4. Highest PLS loadings for Bayley test

Outcome	Feature Type	conditioning	Loading
Cognitive	max TE	no conditioning	0.430
Cognitive	mean TE	no conditioning	0.416
Language	ER mother	mHR accel	0.364
Language	ER mother	no conditioning	0.349
Motor Composite	ER mother	mHR decel	0.353
Motor Fine Skills	mean TE	no conditioning	0.381
Motor Gross Skills	max TE	no conditioning	0.367

Language Composite: Selected: SE mother (mHR decel). R^2(adj) = 0.116. Stress main effect: β = -13.4, p = 0.051 (marginal).

Motor Composite: Selected: max TE (no conditioning), SE mother (conditioned on fHR accel). R^2(adj) = 0.082. TE features selected but not individually significant.

Motor Fine Skills: Selected: SE fetus (conditioned on mHR accel), max TE (no conditioning). R^2(adj) = 0.007. No significant predictors.

Even with parsimonious models (maximum three features), predictive performance remained poor, and individual features were not statistically significant, reinforcing the severe underpowering of multivariate approaches in this sample.

S2.3.6. Integration and Interpretation

While multivariate models failed to achieve reliable predictive performance, the pattern observed in univariate correlations was consistent: TE features were selected by regularized models and ranked highly in feature importance analyses, particularly for motor and cognitive outcomes. PLS loading analysis suggested potential domain specificity: TE features for cognitive/motor outcomes, ER features for language outcomes. However, given the severe underpowering ($n/k \simeq 1.2$) and negative CV-R^2 values, these patterns should be considered exploratory hypotheses requiring replication in adequately powered samples.

In summary, the univariate correlation analyses and the MLM analyses with reduced feature sets provide the most reliable findings for this sample size. Multivariate models would require sample sizes of n $>$ 500 (approximately 10 $\times$ 50 features) for valid inference.

S2.4. Dependence of TE on HR Sampling Rate

Acquiring data at the correct sampling rate is crucial for accurate estimation of the HRV metrics. Here, we test the impact of sampling rates on TE estimates, both with and without conditioning on heart rate dynamics (accelerations and decelerations). We show that TE estimated on full HR time series does not depend on sampling rate, but TE of the conditioned HR does.

After the filtering stage, we resampled the data at the frequency $f_s = 20$Hz. To validate this choice of the sampling rate for our information-theoretical approach, we also explored several other values of the sampling rate f_s: lower values 4Hz, 10Hz, as well as larger values 100Hz and even 1000Hz corresponding to the ECG sampling rate.

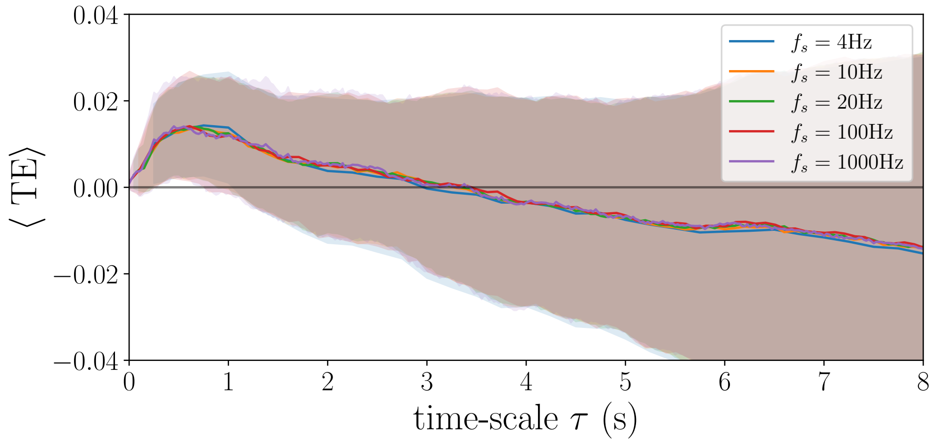

Figure S3. Ensemble-averaged ⟨TE⟩ as a function of the time-scale τ. The shaded areas correspond to the dispersion around the average due to inter-individual variability and are measured as the standard deviation across the cohort. Both the ensemble-averaged TE and its dispersion do not depend on the sampling frequency.

Figure S3 shows the evolution with the timescale τ of the ensemble-averaged $\langle \text{TE} \rangle$, measured by averaging the net TE over the entire cohort. We observe that whatever the sampling frequency f_s used in the pre-processing, the evolution of $\langle \text{TE} \rangle$ with the timescale τ is the same. For all other results in this article, we selected $f_s = 20$ Hz, but we note that 10Hz or even 4Hz is sufficient.

S3. Supplementary Discussion

S3.1. Quantifying Coupling Strength: Mathematical Details

Our MLM analysis revealed that mHR decelerations exert substantially stronger influence on fHR complexity than any other physiological state. This finding arises from the mathematical relationship between the MLM beta coefficients, which may not be immediately apparent from examining the figures alone.

The conditioning framework MLM yields two critical coefficients:

1. Baseline (no conditioning): β = +0.206, p $<$ 0.001 — representing fetal entropy when measured independently of conditioning;
2. Maternal deceleration conditioning: β = -0.123, p = 0.012 — representing entropy change during maternal bradycardic events.

The ratio of these coefficients quantifies proportional coupling strength: 0.123 / 0.206 = 0.597 $\approx$ 60%. This means the maternal deceleration coupling effect (β = -0.123) captures 60% of the dynamic range established by the no-conditioning baseline (β = +0.206). This quantifies how strongly maternal bradycardic state constrains fHR complexity relative to the available entropy variation in our analysis.

This 60% describes the ratio of beta coefficients (coupling strength), not a 60% reduction in actual entropy rate values. The entropy rate decreases by 0.123 units during maternal decelerations; this magnitude represents 60% of the 0.206-unit baseline coefficient, establishing maternal decelerations as the strongest coupling condition detected in our entire analysis.

The coupling strength ratio quantifies the effect relative to the baseline variation, providing a normalized measure of coupling intensity that facilitates comparison across different physiological states. This represents a fundamental coupling mechanism conserved across all pregnancies—both stressed and control groups exhibit this profound entropy reduction during maternal decelerations. This stress invariance is essential for interpreting the findings in the context of past studies.

The profound asymmetry in coupling strength—with maternal decelerations exerting substantially greater influence than maternal accelerations or fetal states—raises an important physiological question: Why should maternal HR decelerations be especially potent in constraining fetal HR dynamics? This question is discussed in the main text.

S3.1.1. Reconciling the 60% Coupling with Stress-Sensitive FSI

Our previous work using bivariate phase-rectified signal averaging (bPRSA) demonstrated that the Fetal Stress Index (FSI) was significantly higher in stressed pregnancies [10]:

- Stressed group: FSI = 0.43 (0.18-0.85), showing fetal HR decreases during maternal decelerations;
- Control group: FSI = 0.00 (-0.49-0.18), fetuses remained "stable" during maternal decelerations.

That is, a higher FSI implies a stronger fHR response, which indicates higher stress levels. This causes an apparent contradiction: How can the current study report a stress-invariant 60% coupling strength reduction when [10] reported a stress-sensitive FSI?

We posit that this contradiction is resolved when one considers the different aspects of coupling. FSI captures the magnitude of the fetal HR decrease and is stress-sensitive, while conditioned entropy captures fHR complexity reduction and is stress-invariant. The entropy reduction (60%) and the fHR response magnitude (FSI) capture complementary but distinct physiological phenomena:

1. *Entropy reduction* (current study - universal): Quantifies how predictable/constrained fetal HR becomes during maternal decelerations. This occurs in ALL pregnancies and represents a fundamental maternal-fetal coordination.
2. *FSI magnitude* ([10] - stress-sensitive): Quantifies how strongly the fHR actually decreases during maternal decelerations. This response magnitude is modulated by stress.

The 60% entropy reduction represents the "coupling channel"—a universal communication pathway between mother and fetus that exists regardless of stress. What stress modulates is the signal transmitted through this channel—specifically, the amplitude and directionality of fetal HR responses.

S3.2. The Three-Layer Conditioning Framework

Our conditioning framework represents a conceptual advance in characterizing physiological coupling. Traditional bivariate coupling measures, including bPRSA, typically compare signals under different conditions or quantify overall coupling strength. The conditioning approach systematically dissects:

1. Univariate properties: Baseline signal complexity independent of coupling
2. Self-conditioned properties: How signals change during their own events
3. Cross-conditioned properties: How signals change during the other signal's events (true bivariate coupling)

This hierarchical approach is critical for interpretation. A change in fetal heart rate entropy during maternal decelerations could reflect: (a) the fetus happening to have its own decelerations simultaneously (self-conditioning), or (b) genuine constraint imposed by the maternal state (cross-conditioning). By quantifying both, we isolate true bivariate coupling from coincidental state matching.

The substantial coupling effect under cross-conditioning—with coupling strength of approximately 60% during maternal decelerations—demonstrates that fHR complexity is genuinely constrained by maternal physiological states, not merely correlated due to independent fluctuations. This mechanistic specificity goes beyond correlation to reveal directional physiological influence.

S4. Supplementary Box: Understanding Beta Coefficient Derivation

For readers unfamiliar with MLM coefficient interpretation:

Mixed linear models (MLM) with categorical predictors (like "Conditioning" with levels: none, mother_accel, mother_decel, fetus_accel, fetus_decel) use reference level coding. One category serves as the baseline (reference = 0), and beta coefficients represent differences from that reference.

In our MLM:

- Reference level: fetus_accel conditioning (β = 0 by definition)
- No conditioning: β = +0.206 (entropy is 0.206 units higher than fetus_accel reference)
- Mother_decel: β = -0.123 (entropy is 0.123 units lower than fetus_accel reference)

The 60% calculation uses the no-conditioning coefficient as the baseline because it represents the maximal entropy state (independent measurement without event-specific conditioning). The maternal deceleration effect (β = -0.123) is expressed as a proportion of this maximal entropy:

$$\textbf{Coupling strength} = \frac{|\ \beta \text{ maternal decelerations }|}{\beta \text{ no conditioning}} = \frac{0.123}{0.206} = 0.597 \approx 60\%$$

This quantifies how much of the "available" entropy (i.e., dynamic range or the difference between the unconditioned state and the reference) is eliminated by maternal deceleration conditioning—revealing the magnitude of the constraint imposed by mHR decelerations on fHR dynamics. It's a measure of relative coupling strength, not absolute entropy percentage change.

Alternative calculation using predicted values:

- Predicted entropy (no conditioning): 0.206 + reference_value
- Predicted entropy (mother_decel): -0.123 + reference_value
- Difference: 0.206 - (-0.123) = 0.329
- Proportional reduction: 0.123 / 0.206 = 60%

Both approaches yield the same conclusion: maternal decelerations reduce fetal entropy by 60% relative to the unconditioned baseline, quantifying the profound asymmetric coupling in maternal-fetal HR dynamics.

References

1. F, M.; Y, K.; M, P.; AH, K. Quantifying the Interactions between Maternal and Fetal Heart Rates by Transfer Entropy. *PLoS ONE* **2015**, *10*, e0145672. https://doi.org/10.1371/journal.pone.0145672.
2. Piskorski, J.; Guzik, P. Asymmetric properties of long-term and total heart rate variability. *Med Biol Eng Comput* **2011**, *49*, 1289—-1297. https://doi.org/10.1007/s11517-011-0834-z.
3. Spilka, J.; Roux, S.G.; Garnier, N.B.; Abry, P.; Goncalves, P.; Doret, M. Nearest-Neighbor Based Wavelet Entropy Rate Measures for Intrapartum Fetal Heart Rate Variability. *Conf. Proc. IEEE Eng. Med. Biol. Soc. (EMBC)* **2014**, pp. 2813–2816. https://doi.org/10.1109/EMBC.2014.6944208.
4. Granero-Belinchon, C.; Roux, S.G.; Abry, P.; Doret, M.; Garnier, N.B. Information Theory to Probe Intrapartum Fetal Heart Rate Dynamics. *Entropy* **2017**, *19*, 640. https://doi.org/10.3390/e19120640.
5. Granero-Belinchon, C.; Roux, S.G.; Abry, P.; Garnier, N.B. Probing High-Order Dependencies With Information Theory. *IEEE Trans. Signal Process.* **2019**, *67*, 3796–3805. https://doi.org/10.1109/TSP.2019.2920472.
6. Schreiber, T. Measuring Information Transfer. *Phys. Rev. Lett.* **2000**, *85*, 461–464.
7. Avci, R.; Escalona-Vargas, D.; Siegel, E.; Lowery, C.; Eswaran, H. Coupling Analysis of Fetal and Maternal Heart Rates via Transfer Entropy Using Magnetocardiography. In Proceedings of the 40th Annual International Conference of the IEEE Engineering in Medicine and Biology Society (EMBC), Honolulu, HI, USA, 2018, pp. 1–4. https://doi.org/10.1109/EMBC.2018.8513053.

8. TJ, N.; M, B.; R, J.; M, M.; van der Ven M.; van der Woude DAA.; SG, O.; van Laar, J.; Vullings, R. Evidence and clinical relevance of maternal-fetal cardiac coupling: A scoping review. *PLoS ONE* **2023**, *18*, e0287245. https://doi.org/10.1371/journal.pone.0287245.
9. Kraskov, A.; Stogbauer, H.; Grassberger, P. Estimating Mutual Information. *Phys. Rev. E* **2004**, *69*, 066138. https://doi.org/10.1103/PhysRevE.69.066138.
10. Lobmaier, S.M.; Mensing, M.; Müller, A.; Lorenz, E.; Bechtold-Dalla Pozza, S.; Hüner, B.; Kuschel, B.; Nennstiel, U.; Wimberger, P.; Antonelli, M.C. Fetal Heart Rate Variability Responsiveness to Maternal Stress, Non-invasively Detected from Maternal Transabdominal ECG. *Archives of Gynecology and Obstetrics* **2020**, *301*, 405–414. https://doi.org/10.1007/s00404-019-05390-8.

S6. Supplementary Tables

Table S5. Complete MLM results for acceleration/deceleration analysis

Effect	β	SE	p-value	Sig
Event_Type (deceleration)	-0.0606	0.0028	<0.001	***
HR_Source(maternal) × Event_Type(decel)	+0.0282	0.0028	<0.001	***
HR_Source (maternal)	-0.0109	0.0028	<0.001	***
Sex (male)	-0.0026	0.0028	0.361	ns
Stress (stressed)	+0.0007	0.0027	0.802	ns
Sex × Stress	-0.0001	0.0029	0.985	ns
Sex × HR_Source	+0.0027	0.0029	0.342	ns
Sex × Event_Type	+0.0014	0.0029	0.626	ns
Stress × HR_Source	-0.0005	0.0028	0.865	ns
Stress × Event_Type	-0.0013	0.0028	0.651	ns

Note. Model: Fraction S̃ex × Stress × HR_Source × Event_Type + (1 | Patient_ID) REML estimation, n=118 patients, 472 observations

Table S6. Complete MLM results for entropy rate conditioning analysis

Effect	β	SE	p-value	Sig	Analysis Layer
Conditioning Effects					
Conditioning (none)	+0.2061	0.0351	<0.001	***	Univariate baseline
Conditioning (mother_decel)	-0.1228	0.0491	0.012	*	Cross-conditioned (bivariate)
Conditioning (fetus_decel)	-0.0816	0.0424	0.054	†	Cross-conditioned (bivariate)
Conditioning (mother_accel)	-0.0335	0.0490	0.494	ns	Cross-conditioned (bivariate)
Metric & Signal					
Metric (hmean)	-0.1172	0.0515	0.023	*	-
HR_Source (mother)	+0.0534	0.0337	0.113	ns	-
Demographic					
Sex (male)	-0.1058	0.0784	0.177	ns	-
Stress (stressed)	-0.0852	0.0559	0.128	ns	-
Sex × Stress	+0.1079	0.0886	0.223	ns	-

Note. † $p < 0.10$ (marginal trend). Model: Value S̃ex × Stress × Metric × HR_Source × Conditioning + (1 | Patient_ID). REML estimation, n=118 patients, 1,006 observations

Table S7. Entropy rate versus sample entropy: data quality comparison

Conditioning Type	Entropy Rate (Hmax/Hmean)	Sample Entropy
Total observations	1,006	2,348
Obs per patient	8.5	19.9
fetus_full	100% non-zero	100% non-zero
mother_full	100% non-zero	100% non-zero
fetus_fHR_decel	1̃00% non-zero	99.2% non-zero
mother_fHR_decel	1̃00% non-zero	99.2% non-zero
fetus_mHR_accel	1̃00% non-zero	99.2% non-zero
mother_mHR_accel	1̃00% non-zero	99.2% non-zero
fetus_mHR_decel	1̃00% non-zero	100% non-zero
mother_mHR_decel	1̃00% non-zero	100% non-zero

Table S8. Complete correlation matrix: Transfer Entropy features vs. cortisol

Feature	r	p	Significance
Max TE fHR (all)	+0.083	0.437	ns
Max TE fHR (accel)	+0.250	0.019	*
Max TE fHR (decel)	+0.271	0.011	*
Max TE mHR (all)	+0.083	0.437	ns
Max TE mHR (accel)	+0.287	0.007	**
Max TE mHR (decel)	+0.315	0.003	**
Mean TE fHR (all)	+0.057	0.594	ns
Mean TE fHR (accel)	+0.212	0.047	*
Mean TE fHR (decel)	+0.207	0.053	ns
Mean TE mHR (all)	+0.057	0.594	ns
Mean TE mHR (accel)	+0.221	0.038	*
Mean TE mHR (decel)	+0.217	0.042	*

Note. ns = not significant; * $p < 0.05$; ** $p < 0.01$.

Table S9. Transfer entropy MLM results: Conditioning and demographic effects

Effect	β	SE	p-value	Significance
Conditioning & Metric				
TE metric (Mean)	-0.0773	0.0073	<0.001	***
HR event (None/Baseline)	-0.0374	0.0072	<0.001	***
TE metric × HR event (None)	+0.0636	0.0102	<0.001	***
Demographic Effects				
Stress (stressed)	+0.0233	0.0105	0.026	*
Sex × Stress	-0.0425	0.0164	0.009	**
Sex × Stress × HR event (None)	+0.0367	0.0149	0.014	*
Non-Significant				
Sex (male)	+0.0176	0.0111	0.113	ns
Conditioning source (maternal)	+0.0050	0.0073	0.488	ns

Note. Model specification: TE value ∼ Sex × Stress × TE_type × Conditioning_source × HR_event + (1 | Patient_ID), REML estimation, n=118 patients.

S7. Supplementary Figures

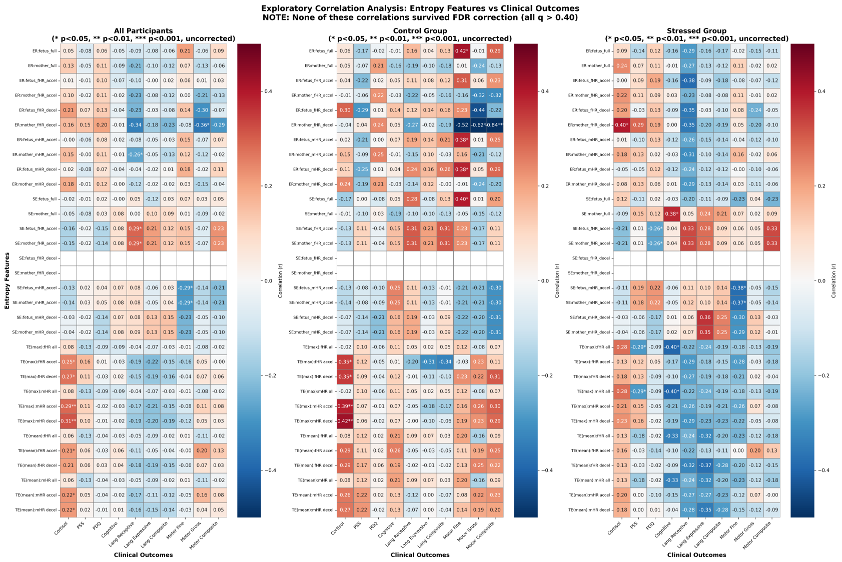

Figure S4. Exploratory correlation analysis between entropy features and clinical outcomes.

Note. Heatmaps show correlation coefficients (r) for all participants (left), control group (center), and stressed group (right). Asterisks indicate uncorrected significance (* p<0.05, ** p<0.01, *** p<0.001). **CRITICAL**: None of these correlations survived False Discovery Rate correction (all q > 0.40); all findings are exploratory and hypothesis-generating only.

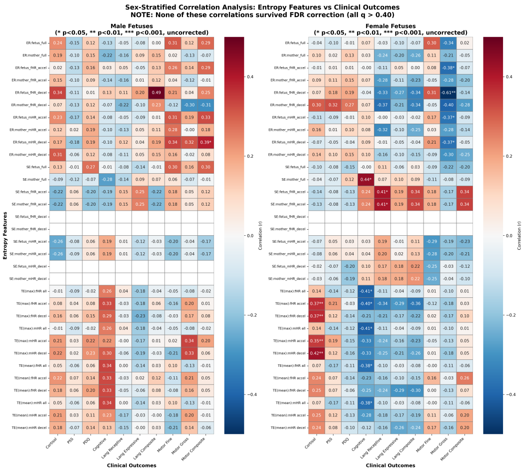

Figure S5. Sex-stratified sample entropy and entropy rate correlations to outcomes

Note. Heatmaps show correlation coefficients (r) for male fetuses (n=49, left), and female fetuses (n=69, right). Asterisks indicate uncorrected significance (* p<0.05, ** p<0.01, *** p<0.001). **CRITICAL**: None of these correlations survived False Discovery Rate correction (all q > 0.40); all findings are exploratory and hypothesis-generating only.

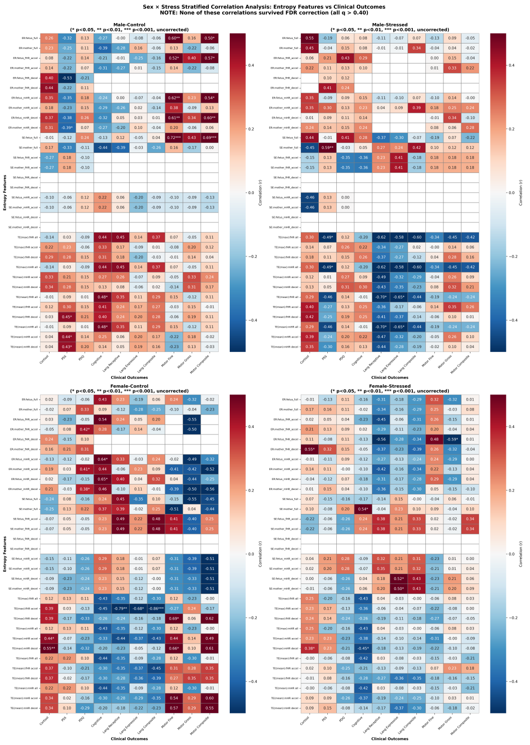

Figure S6. Sex × Stress stratified analysis of sample entropy and entropy rate correlations to outcomes

Note. Exploratory sex × stress interaction analysis of entropy-outcome correlations. Four-panel heatmap (2×2 grid) showing correlation coefficients (r) for: Male-Control (top-left, n=30), Male-Stressed (top-right, n=19), Female-Control (bottom-left, n=32), and Female-Stressed (bottom-right, n=39). **CRITICAL**: None of these correlations survived False Discovery Rate correction (all q > 0.40); all findings are exploratory and hypothesis-generating only.

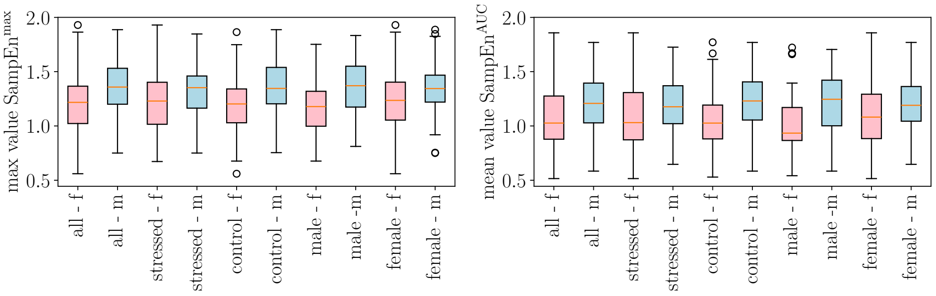

Figure S7. Typical dependence of the maximal value $\text{SampEn}^{\text{max}}$ of the sample entropy (left) as well as of its mean value $\text{SampEn}^{\text{AUC}}$ (right) in the range $[0.5 - 2.5]$s for the mHR (blue - m) and the fHR (pink - f) across the cohort, as well as in each subgroup (stressed or control, female or male foetus).

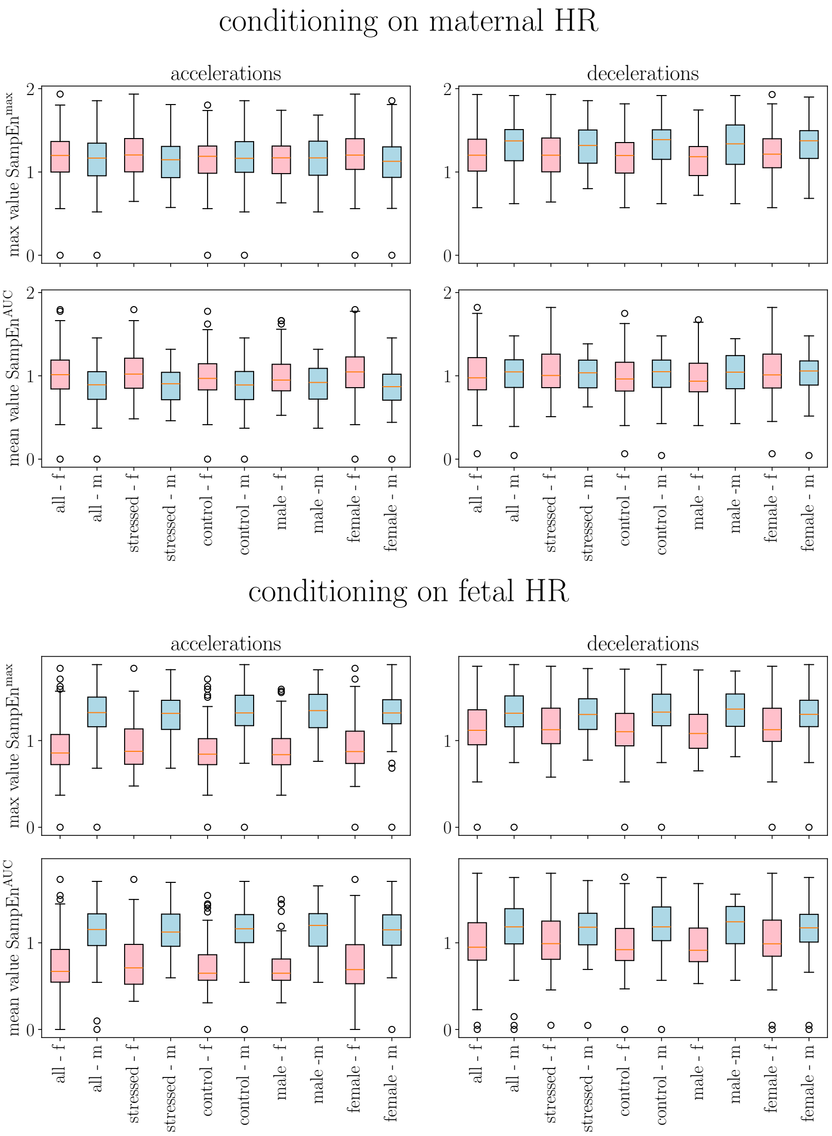

Figure S8. Same as Fig. S7 but when considering either accelerations of decelerations, computed on either maternal or fetal HR.
